# Artificial Intelligence-Guided Extracellular Vesicle Platforms for Diagnosis and Treatment of Chronic Kidney Disease: A Scoping Review and Future Directions

**DOI:** 10.3390/medicina62101878

**Published:** 2026-09-28

**Authors:** Kumar Digvijay, Henrik Birn, Claudio Ronco

**Affiliations:** 1 Department of Renal Medicine, Aarhus University Hospital, 8200 Aarhus, Denmark; 2 IRRIV—International Renal Research Institute, 36100 Vicenza, Italy; cronco@goldnet.it; 3 Department of Nephrology, Dialysis and Transplantation, San Bortolo Hospital, 36100 Vicenza, Italy; 4 Università degli Studi di Padova, 35122 Padova, Italy

**Keywords:** artificial intelligence, extracellular vesicles, chronic kidney disease, biomarkers, drug delivery, machine learning, precision medicine, exosomes, microvesicles

## Abstract

*Background and Objectives*: Chronic kidney disease (CKD) affects roughly 788 million adults worldwide according to the Global Burden of Disease (GBD) 2023 estimate. Extracellular vesicles (EVs) are promising sources of biomarkers and therapeutic carriers, while artificial intelligence (AI) offers tools for high-dimensional EV analysis and computational design. *Materials and Methods*: We conducted a scoping review using a structured PubMed/MEDLINE search, run on 7 September 2026, and PRISMA-ScR reporting principles. Eligible studies applied AI/ML to EV-derived or closely related kidney-disease data. The review was not prospectively registered. The formal scoping evidence set was used to map kidney EV–AI studies focused on diagnosis, prognosis, and biomarker discovery; EV engineering, therapeutic design, manufacturing, and physiologically based pharmacokinetic (PBPK) modeling were synthesized separately as contextual and future-oriented literature identified through non-systematic contextual literature identification. Predictive models were critically appraised using a PROBAST-informed framework. *Results*: Nine reports were retained in the formal PRISMA evidence set. Direct kidney EV–AI evidence was limited and heterogeneous, with small cohorts and variable validation strategies. Several studies reported encouraging discrimination, but external validation, calibration, standardized EV workflows, and prospective clinical utility were uncommon. Contextual literature suggests potential roles for AI in EV engineering, manufacturing, and PBPK-informed therapeutic development, but these applications remain investigational in CKD. *Conclusions*: AI–EV integration is a promising research framework rather than a clinically validated platform. Translation will require standardized EV methods, adequately powered multicentre studies, independent validation, clinically relevant operating thresholds, prospective utility studies, and explicit manufacturing and regulatory strategies.

## 1. Introduction

The Global Burden of Disease Study 2023 estimated that 788 million adults aged 20 years or older were living with CKD in 2023, corresponding to a global age-standardized prevalence of 14.2% [1]. This estimate provides the epidemiological baseline used throughout this review; older estimates are discussed separately where relevant.

By 2023, CKD had become the ninth leading cause of death globally [1]. The burden is not distributed evenly: North Africa and the Middle East had the highest age-standardized prevalence (18.0%), followed by South Asia (15.8%) and sub-Saharan Africa (15.6%) [1], reflecting interacting genetic, environmental, metabolic, and socioeconomic determinants.

Important limitations remain in the diagnosis and management of CKD. Serum creatinine-based estimated glomerular filtration rate (eGFR) and the urinary albumin-to-creatinine ratio (UACR) remain central to clinical assessment, but both have limitations for detecting early kidney injury, characterizing disease heterogeneity, and identifying patients at risk of rapid progression. Biomarkers that detect kidney injury earlier, improve prediction of individual disease trajectories, and support more personalized therapeutic decision-making are therefore needed. Therapies that target key mechanisms of CKD progression, including fibrosis, chronic inflammation, and endothelial dysfunction, while minimizing off-target effects associated with systemic drug delivery are also needed.

Extracellular vesicles (EVs) have attracted considerable interest as tools for biomarker discovery and therapeutic delivery. EVs are heterogeneous membrane-enclosed particles that can be characterized by operational features such as size, density, molecular composition, and cellular origin. Terms such as exosome and microvesicle should be used cautiously because specific biogenesis is often not demonstrated experimentally. EV cargo includes proteins, lipids, metabolites, and nucleic acids, including miRNAs, mRNAs, and other non-coding RNAs [2], and may reflect the molecular state of the cells from which the vesicles originate. In the kidney, urinary extracellular vesicles (uEVs) provide a minimally invasive source of molecular information from renal and urinary tract cells [3].

Artificial Intelligence–Virtual Extracellular Vesicles (AIVEVs) have recently been proposed as an umbrella concept integrating artificial intelligence with EV-based biomarker discovery, characterization, engineering, and therapeutic development, rather than as a standardized technical or regulatory category [4]. In this review, “EV–AI” refers to studies that apply artificial intelligence or machine-learning methods to EV-derived data, whereas “AIVEV” denotes the broader conceptual framework.

This review critically evaluates the current state of the AIVEV framework in the context of CKD. We examine recent advances in AI-enabled EV biomarker discovery, computational approaches to therapeutic EV engineering, the potential role of physiologically based pharmacokinetic (PBPK) modeling in renal drug delivery, and the translational, manufacturing, regulatory, and validation requirements that must be addressed before AIVEV-based strategies can be considered for routine clinical application.

## 2. Materials and Methods

Protocol and registration: This scoping review was reported in accordance with the PRISMA Extension for Scoping Reviews (PRISMA-ScR) [5], with reporting additionally cross-checked against the PRISMA 2020 checklist [6]. A completed PRISMA 2020 checklist is supplied as a separate reporting file, the PRISMA-ScR checklist is provided in the Supplementary Materials, and the study-selection process is presented in the PRISMA 2020 flow diagram. The review was not prospectively registered, and no review protocol was deposited before conduct of the review.

Screening and data extraction were structured according to study population, EV source and pre-analytical workflow, predictor modality, AI method, outcome definition, validation strategy, performance metrics, reporting of calibration and clinical utility, and translational limitations. The archived review materials do not permit reliable reconstruction of the number of reviewers who performed title/abstract screening, full-text eligibility assessment, data extraction, or the PROBAST-informed appraisal, nor do they document duplicate independent review at these stages. Accordingly, no retrospective claim of duplicate independent screening, extraction, or risk-of-bias assessment is made. No automation tools were used for screening or data extraction. Given the heterogeneity of the included literature and the absence of sufficiently comparable data for quantitative pooling, the findings were synthesized narratively and thematically.

**Eligibility criteria:** Eligible records included peer-reviewed original research and systematic or scoping reviews that applied an artificial intelligence (AI) or machine-learning (ML) method directly to EV-based diagnosis, biomarker discovery, EV characterization, EV engineering, therapeutic EV design, or a closely related nephrology/EV methodological problem, and that provided sufficient methodological or performance information for critical appraisal. We excluded conference abstracts without full-text publications, case reports, non-English-language records for which a translation was unavailable, studies unrelated to EVs, and studies without an AI/ML component.

**Information sources and search strategy:** PubMed/MEDLINE was the primary bibliographic database, supplemented by backward citation tracking of relevant reviews and primary studies. The PubMed/MEDLINE search was conducted on 7 September 2026 without a lower publication-date restriction. The core search strategy was:

(“extracellular vesicle”[Title/Abstract] OR “extracellular vesicles”[Title/Abstract] OR exosome*[Title/Abstract] OR microvesicle*[Title/Abstract] OR “small extracellular vesicles”[Title/Abstract]) AND (“artificial intelligence”[Title/Abstract] OR “machine learning”[Title/Abstract] OR “deep learning”[Title/Abstract] OR transformer*[Title/Abstract] OR “generative AI”[Title/Abstract] OR “generative model”[Title/Abstract] OR “graph neural network”[Title/Abstract] OR “random forest”[Title/Abstract] OR XGBoost[Title/Abstract] OR “digital twin*”[Title/Abstract] OR “federated learning”[Title/Abstract]) AND (“chronic kidney disease”[Title/Abstract] OR CKD[Title/Abstract] OR “kidney disease”[Title/Abstract] OR renal[Title/Abstract]) AND (diagnos*[Title/Abstract] OR prognos*[Title/Abstract] OR biomarker*[Title/Abstract] OR therapeut*[Title/Abstract] OR “drug delivery”[Title/Abstract] OR manufacturing[Title/Abstract] OR pharmacokinetic*[Title/Abstract] OR translation[Title/Abstract]).

Review framework and question: We conducted a structured scoping review to address the following question: What is the current evidence for applying AI or ML methods to EV-derived data in kidney disease diagnosis, prognosis, and biomarker discovery, and what methodological limitations affect the validity and translation of these studies?

Study selection: The archived PubMed/MEDLINE export contained 30 records. Following title and abstract screening, nine reports were selected for full-text assessment, all of which met the eligibility criteria and were retained for qualitative synthesis. All nine retained reports concerned diagnosis, prognosis, biomarker discovery, or closely related EV-associated computational analysis; none constituted a clinical study of AI-guided EV engineering, PBPK-guided EV dosing, or therapeutic EV design. Study-level characteristics and evidence roles for the nine retained reports are provided in Supplementary Table S3. Accordingly, the formal scoping evidence set supports the diagnostic and biomarker-focused synthesis, whereas the engineering and therapeutic sections are explicitly presented as contextual and future-oriented narrative synthesis.

Supplementary identification and reconciliation: Contextual literature on computational methods, bioengineering, federated learning, digital twins, manufacturing, and PBPK modeling was identified narratively through backward citation tracking and focused topic searches to support the future-oriented discussion; this contextual literature was not treated as a systematically identified evidence set and was not included in the PRISMA denominator. Comprehensive searches of Scopus, Web of Science, Embase, and IEEE Xplore were not performed. Separately, a seven-row screening file was reconciled against the PubMed/MEDLINE export and yielded no additional eligible studies: two rows duplicated studies already identified in PubMed/MEDLINE, one corresponded to a review authored by members of the present author group and was excluded from the evidence set, and four contained invalid or mismatched PMIDs. Accordingly, the formal PRISMA study-selection counts are based on the documented 30-record PubMed/MEDLINE export.

**Search limitations:** Because the primary database search was restricted to PubMed/MEDLINE, relevant engineering or computational studies indexed exclusively in Scopus, Web of Science, Embase, IEEE Xplore, or other databases may have been missed. This limitation should be considered when interpreting the comprehensiveness of the evidence identified.

Supplementary reporting and risk-of-bias assessment: The review is reported in accordance with the PRISMA extension for Scoping Reviews (PRISMA-ScR) [5], with additional reporting cross-checked against PRISMA 2020 [6]. A completed PRISMA-ScR checklist is provided in Supplementary Table S1, and a completed PRISMA 2020 checklist is supplied as a separate reporting file. The study-selection process is presented in a PRISMA 2020 flow diagram. A PROBAST-informed appraisal, cross-checked against PROBAST + AI, was applied to all nine retained reports containing predictive, classification, or machine-learning biomarker analyses, while recognizing that several were exploratory biomarker-discovery studies rather than formal clinical prediction-model development studies [7,8]. Three non-EV comparator studies used as methodological benchmarks were appraised separately. Domain-level judgments are provided in Supplementary Table S2, the nine retained reports are summarized in Supplementary Table S3, and the screening reconciliation is provided in Supplementary Table S4.

Synthesis approach: The formal PRISMA evidence set was synthesized narratively because the nine retained reports were heterogeneous in kidney-disease setting, EV source, analytical platform, predictor type, and validation strategy. Quantitative pooling, meta-analysis, subgroup analysis, sensitivity analysis, formal assessment of reporting bias, and certainty-of-evidence grading were not undertaken because these procedures were not appropriate to the scoping objective and heterogeneous evidence map. Sections addressing EV engineering, therapeutic design, PBPK modeling, manufacturing, federated learning, and digital twins are clearly distinguished as contextual narrative synthesis based on targeted supplementary literature and should not be interpreted as findings from the nine-study PRISMA evidence set. The PRISMA 2020 flow diagram reports the documented primary database-screening process; the supplementary reconciliation is reported separately because it yielded no additional eligible studies and therefore did not alter the primary PRISMA denominator.

## 3. Results

### 3.1. Global CKD Burden and Unmet Needs

The global burden of CKD provides important context for evaluating the potential relevance of AIVEV-based approaches. The Global Burden of Disease Study 2023 estimated that 788 million adults aged 20 years or older were living with CKD in 2023, corresponding to an age-standardized prevalence of 14.2% [1]. These current estimates are used as the principal epidemiological reference in this review. Key epidemiological estimates and regional differences in CKD burden are summarized in Table 1 and illustrated alongside the conceptual AIVEV framework in Figure 1.

The rising prevalence of CKD is driven by multiple factors, including population aging and increasing rates of obesity, diabetes, and hypertension. These challenges disproportionately affect low- and middle-income countries, where access to kidney replacement therapy remains limited. This disparity is particularly important because populations with a substantial need for early detection and disease-modifying interventions may also have limited capacity to deliver these strategies at scale [11].

**Figure 1:**
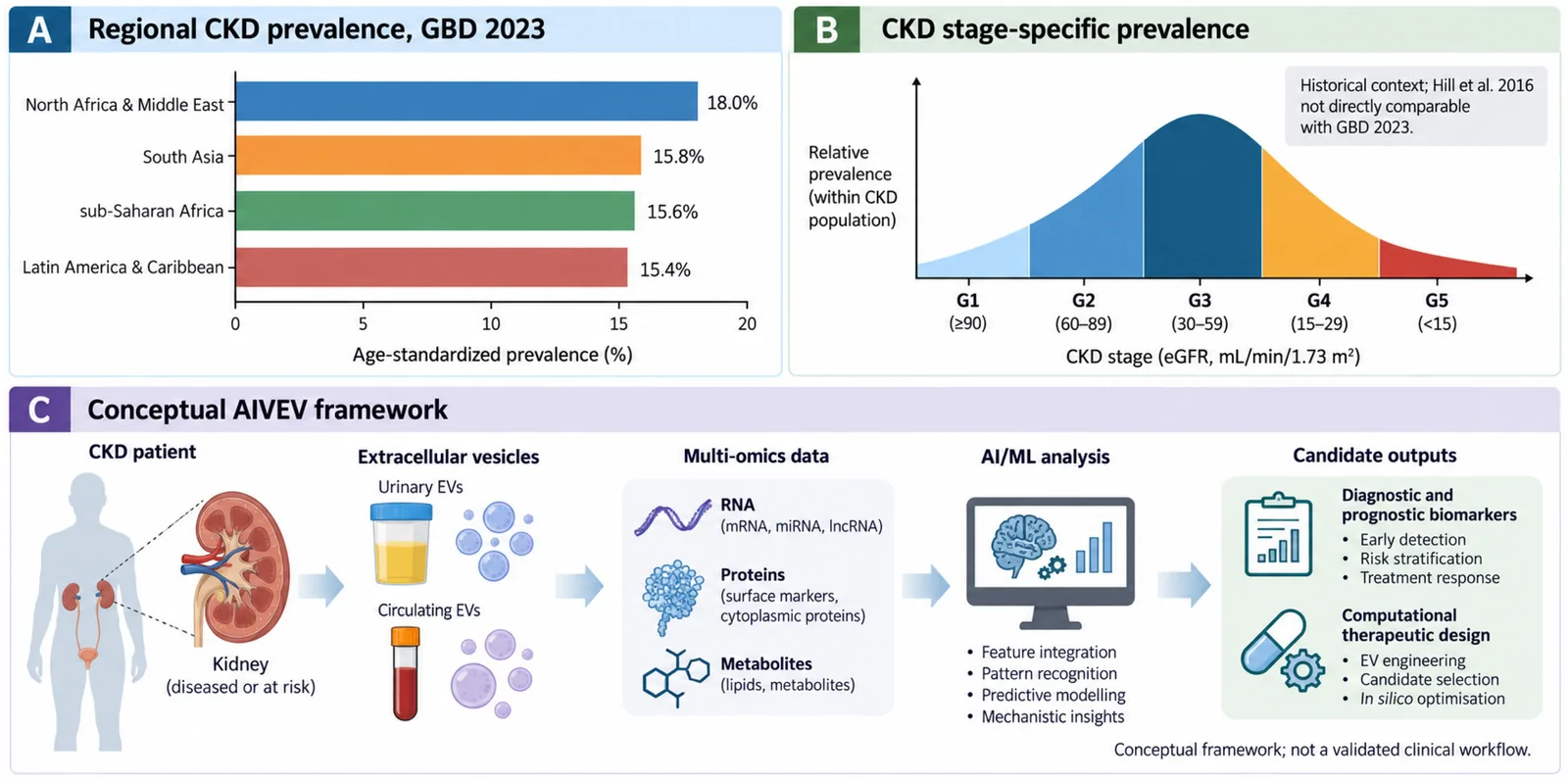
Global CKD burden and conceptual AIVEV framework. (**A**) Age-standardized CKD prevalence in the four highest-burden GBD 2023 super-regions: North Africa and the Middle East (18.0%), South Asia (15.8%), sub-Saharan Africa (15.6%), and Latin America and the Caribbean (15.4%) [1]. (**B**) Schematic CKD stage distribution shown only as historical conceptual context based on the stage-specific framework summarized by Hill et al. [12]; no mortality series or new quantitative estimate is presented. (**C**) Conceptual AIVEV framework developed by the authors; not a validated clinical workflow.

The documented study-selection process is presented in the PRISMA 2020 flow diagram (Figure 2). The primary PubMed/MEDLINE search identified 30 records. After title/abstract screening, 21 records were excluded and nine reports proceeded to full-text assessment; all nine met the eligibility criteria and were retained for qualitative synthesis. No full-text report was excluded. The separate seven-row supplementary reconciliation yielded no additional eligible studies and therefore did not alter the primary PubMed/MEDLINE denominator or the nine-study formal evidence set.

Current CKD management relies substantially on renin–angiotensin–aldosterone system inhibitors, sodium–glucose cotransporter 2 (SGLT2) inhibitors, and mineralocorticoid receptor antagonists. Although these therapies can slow CKD progression, they do not completely prevent progressive loss of kidney function. For patients who progress to kidney failure, dialysis and kidney transplantation are associated with substantial clinical, personal, and economic burdens. The development of therapies capable of directly modulating key mechanisms of CKD progression, including fibrotic signaling, impaired tubular repair, and immune dysregulation, therefore remains an important therapeutic priority [13].

### 3.2. Extracellular Vesicles in CKD: Biology and Clinical Relevance

#### 3.2.1. EV Biogenesis and Heterogeneity

EVs constitute a heterogeneous population of membrane-enclosed particles that differ in biogenesis, size, molecular composition, surface characteristics, and biological function. Exosomes originate within the endosomal system through inward budding of the limiting membrane of multivesicular bodies (MVBs) and are released following fusion of MVBs with the plasma membrane. In contrast, microvesicles are generated by outward budding and fission of the plasma membrane. Apoptotic bodies are generally larger vesicular structures released during programmed cell death [14].

This heterogeneity has important implications for CKD research. EVs originating from different renal cell types, including podocytes, tubular epithelial cells, endothelial cells, and interstitial fibroblasts, may carry molecular cargo that reflects the biological state of their cells and tissues of origin. However, overlap in the physical and biochemical properties of EV subpopulations, lipoproteins, and protein aggregates complicates their isolation, purification, and characterization [15,16]. Recent exosome-focused reviews similarly identify differences in isolation and characterization methods as important sources of pre-analytical and downstream variability in kidney-disease EV studies [17].

#### 3.2.2. EVs as Diagnostic Biomarkers in CKD

EVs have attracted increasing interest as potential diagnostic and prognostic biomarkers in CKD. Urinary extracellular vesicles (uEVs) are particularly relevant because they can be obtained non-invasively and contain molecular cargo derived from cells throughout the nephron and urinary tract. Representative urinary and EV-associated biomarkers investigated in CKD and related kidney diseases are summarized in Table 2.

Several listed biomarkers have substantial supporting evidence as urinary or circulating biomarkers; however, only a subset have been specifically localized to, or shown to be enriched in, urinary extracellular vesicles. Table 2 therefore distinguishes direct EV evidence from broader urinary-biomarker and comparator evidence. Encapsulation within the EV lipid bilayer can protect miRNAs from extracellular RNase-mediated degradation, potentially improving stability during sample processing, storage, and transport. EV-associated surface proteins may likewise provide relatively stable targets for affinity-based detection. Despite these potential advantages, clinical translation remains limited by pre-analytical variability, the lack of standardized EV isolation and characterization protocols, and insufficient validation in large, diverse, independent patient cohorts [29].

#### 3.2.3. Pre-Analytical Variability as a Confounder of Downstream AI Models

The choice of EV isolation method is not a neutral technical consideration, because it can substantially influence the biological and analytical features available to downstream AI/ML models. Ultracentrifugation, size-exclusion chromatography, polymer-based precipitation, and microfluidic approaches can recover different and only partially overlapping EV populations, with differences in yield, size distribution, purity, and protein and RNA profiles. Consequently, a model trained on EV data generated using one isolation workflow may capture platform- or protocol-specific features rather than disease-associated biological signals and may generalize poorly to data generated using different isolation procedures, equipment, laboratories, or operators. Pre-analytical variability therefore represents an important potential threat to the external validity and reproducibility of EV-based predictive models, in addition to the limitations associated with small sample sizes and high-dimensional data discussed in Section 3.3.1.

The MISEV2023 guidelines of the International Society for Extracellular Vesicles provide a framework for improving the standardization and reporting of EV separation and characterization [30]. However, reporting of pre-analytical procedures, EV isolation, and characterization remains variable across the AI/ML studies considered in this review, limiting direct comparison between studies and potentially contributing to poor reproducibility across independent datasets.

#### 3.2.4. EVs as Therapeutic Agents in CKD

Beyond their diagnostic potential, EVs, particularly those derived from mesenchymal stromal cells (MSCs), have demonstrated therapeutic effects in preclinical models of kidney disease. MSC-derived EVs (MSC-EVs) have been reported to exert renoprotective effects through multiple mechanisms, including modulation of inflammation and apoptosis, promotion of angiogenesis and cellular proliferation, attenuation of oxidative stress, and regulation of immune-cell polarization [31].

Recent single-cell RNA-sequencing studies have provided further insight into the cellular and molecular mechanisms underlying these effects. In a model of diabetic kidney disease (DKD), MSC-derived small EVs were reported to attenuate renal fibrosis through delivery of CK1δ/β-TRCP, promoting YAP ubiquitination and degradation and thereby suppressing the profibrotic TGF-β1/Smad2/3/YAP signaling axis [32]. The same study identified fibrosis-associated macrophages as potentially important contributors to DKD progression and reported that MSC-derived small-EV treatment partially restored the distribution of renal cell populations altered during disease progression [32].

Limited clinical evidence has also begun to emerge. In a randomized placebo-controlled trial involving 40 patients with stage III or IV CKD, 20 participants received two doses of cell-free cord blood-derived MSC-EVs (100 μg/kg), while 20 received placebo. During 12 months of follow-up, the MSC-EV group was reported to show improvements in eGFR, serum creatinine, blood urea, urinary albumin-to-creatinine ratio, and inflammatory cytokine profiles, with no significant adverse effects reported [33]. Although these findings provide preliminary evidence supporting the clinical investigation of MSC-EVs, the small sample size and limited evidence base preclude conclusions regarding efficacy, long-term safety, or generalizability. Larger, independently conducted clinical trials with standardized EV characterization and predefined clinical endpoints are required.

### 3.3. Artificial Intelligence in EV Research: Foundational Concepts

AI and ML approaches have become increasingly relevant to EV research because of several methodological challenges, including substantial EV heterogeneity, the high dimensionality of multi-omics data, the complexity of image-based characterization, and the need to develop models that remain robust and generalizable across heterogeneous experimental and clinical settings [34].

#### 3.3.1. The Dimensionality–Sample Size Mismatch

EV multi-omics profiling can generate hundreds to thousands of proteomic, transcriptomic, lipidomic, and metabolomic features per sample, whereas many CKD-focused EV studies, including several evaluated in this review, comprise relatively small cohorts. This high-dimensional setting, in which the number of candidate predictors may substantially exceed the number of observations (*p* ≫ *n*), increases the risk of overfitting. Under these conditions, apparently high internal discrimination, including AUROC estimates, may reflect cohort-specific noise rather than reproducible biological signals, particularly when feature selection, hyperparameter optimization, and performance evaluation are not appropriately nested within cross-validation procedures.

Strategies to mitigate these risks include L1/L2 regularization, dimensionality reduction, prespecification of candidate features, nested cross-validation, independent external validation, and, where appropriate, multicentre or federated-learning approaches [33,35]. However, these safeguards are not consistently reported across the EV–AI studies considered in this review. Accordingly, reported model performance should be interpreted in the context of sample size, predictor dimensionality, feature-selection procedures, validation strategy, calibration, and external validation. These considerations are incorporated into the structured risk-of-bias assessment described below.

Importantly, evidence that machine-learning models perform well using conventional clinical or urinary biomarkers does not, by itself, demonstrate that EV-derived multi-omic features will provide comparable or superior predictive performance. Establishing such a benefit requires direct EV–AI studies with adequate sample sizes, standardized pre-analytical workflows, rigorous model development, and validation in independent populations.

#### 3.3.2. Machine Learning for EV Classification and Biomarker Discovery

Machine-learning methods have been applied to EV-derived molecular and imaging data for classification, feature selection, and biomarker discovery. Table 3 summarizes representative AI/ML approaches relevant to EV research in CKD, together with their principal applications and methodological considerations. Combining complementary analytical approaches may improve discrimination or biological interpretation in selected EV studies; however, evidence of consistent superiority over single-modality approaches remains limited.

Methodological examples from outside nephrology further illustrate the analytical potential of AI-assisted EV profiling but should not be interpreted as direct evidence of performance in CKD. In hepatocellular carcinoma, Li et al. integrated a DNA cascade reaction-triggered individual EV nanoencapsulation (DCR-IEVN) assay with machine-learning classifiers and reported an overall validation-cohort accuracy of 93.3% for distinguishing hepatocellular carcinoma, cirrhosis, and healthy donors [36]. Similarly, total internal reflection fluorescence (TIRF) imaging combined with deep learning has demonstrated high classification performance using single-vesicle miRNA profiling [37]. AI-assisted EV-based liquid-biopsy approaches have also been investigated in other disease settings, including breast cancer, as potential tools for early detection [35]. These studies provide methodological context for EV–AI development but do not establish diagnostic performance or clinical utility in CKD.

**Table 3:** AI/ML Techniques Applied to EV Research in CKD.

AI/ML Approach	Evidence Setting	EV Relationship	Representative Evidence	Interpretive Status
Random forest	CKD progression (non-EV biomarker model)	Not EV-specific	AUC 0.88 internal and 0.87 external at 2 years in a large CKD cohort [38]	Methodological precedent; not an EV model
Random forest/biomarker model	Diabetic kidney disease	Plasma biomarkers + EHR; not EV-specific	AUC 0.77; PPV 61% in high-risk group; NPV 90% in low-risk group [39]	Clinical-utility precedent
Supervised learning	Kidney transplant	Serum/urine EV surface markers	EV signature predicted 1-year renal outcome; small cohort (*n* = 58) with extensive model search [40]	Exploratory EV-specific evidence
Machine learning	Type 2 diabetic nephropathy	Urinary EV RNA	Validation AUC 0.877; independent test AUC 0.824; MYO1C most influential feature [41]	Direct CKD EV–AI evidence; still limited cohort size
Three ML algorithms	Lupus nephritis	Urinary EV RNA	Candidate transcripts prioritized computationally and evaluated in an independent 143-sample validation cohort [42]	Direct kidney EV–AI evidence
Random forest	Lupus nephritis	Urinary EV metabolomics	Three candidate metabolites AUC 0.912, 0.906 and 0.897 [43]	Direct kidney EV–AI evidence; external generalizability not established
CNN/U-Net/SVM and related methods	EV imaging and cancer liquid biopsy	EV imaging or tumor-EV settings	High performance reported in non-kidney EV applications [15,44]	Transferable methodology, not CKD validation
VAE/GNN/transformer concepts	EV engineering and generative design	EV engineering/computational biology	Latent representation, interaction modelling and sequence-learning approaches described in methodological literature [15,42]	Conceptual or preclinical; not clinically validated
RF/LASSO/SVM-RFE	SLE/lupus nephritis	Serum exosome metabolomics	Three-metabolite panel; AUC 0.931 for LN vs. non-renal SLE [45]	Supporting kidney EV–AI evidence; case–control, single study
Six ML algorithms; XGBoost best	Diabetic kidney disease	Genetic/clinical predictors with urinary-exosome GSK3β substudy	ROC 0.877; accuracy 0.825; GSK3B variants ranked among top predictors [46]	Supporting evidence; not a pure EV-derived classifier
WGCNA/ceRNA + ML classifiers	Type 2 diabetic nephropathy	uEV whole-transcriptome profiling	Six candidate RNAs proposed from 24 participants [47]	Exploratory discovery evidence; very small cohort
XGBoost/RF + MR	Diabetic kidney disease	Tissue transcriptomics; EV association via Vesiclepedia	CMAS/RGS10 EV-associated biomarkers; nomogram AUC 0.930 [48]	Supporting computational evidence; not a direct clinical EV assay
Nonlinear SVM/PLS-DA + WGCNA	Pediatric FSGS on peritoneal dialysis	Mesothelial exosome proteomics	Complete separation in 6 FSGS vs. 6 non-FSGS; ANXA13/TIMP1 signals [49]	High-risk exploratory evidence; extremely small cohort

#### 3.3.3. Deep Learning for Single-Vesicle Analysis

Advances in single-vesicle analytical technologies, including nanoparticle tracking analysis (NTA), single-particle interferometric reflectance imaging (SP-IRIS), and high-sensitivity flow cytometry, have substantially increased the resolution and complexity of EV-derived datasets. Deep-learning approaches may be particularly useful for extracting patterns from these high-dimensional data. Autoencoders and variational autoencoders (VAEs), for example, can learn lower-dimensional latent representations of complex datasets and may facilitate the identification of phenotypic patterns and rare EV subpopulations that could be difficult to resolve using conventional analytical approaches [50].

Attention-based transformer models and graph neural networks (GNNs) may offer additional opportunities to integrate heterogeneous EV datasets, identify relationships among molecular features, and prioritize EV subpopulations or candidate biomarkers for further investigation. At present, however, these applications should be regarded primarily as emerging computational strategies rather than validated approaches for CKD. Computationally generated associations and predictions require independent experimental and clinical validation before biological or diagnostic significance can be established [50].

### 3.4. AIVEVs for CKD Diagnosis: From Biomarker Panels to Digital Pathology

#### 3.4.1. Multi-Omics Integration and AI-Enhanced Biomarker Discovery

Integrating multi-omics data with EV analysis is a promising approach to CKD biomarker discovery; however, the principal analytical challenge is whether sample size, data quality, and validation strategies are adequate for the dimensionality of the resulting feature space. Multi-omics integration in nephrology is affected by missing data, batch effects, high dimensionality, platform heterogeneity, and limited model interpretability. In EV-based studies, these challenges may be amplified by pre-analytical variability in biofluid collection and storage, EV isolation, normalization, and vesicle characterization [29,35].

AI and machine-learning approaches may help address this complexity by integrating heterogeneous molecular data and identifying combinations of EV-associated RNAs, proteins, lipids, and metabolites that may be difficult to prioritize using conventional univariate analyses. Feature-selection and dimensionality-reduction methods can reduce the effective number of candidate variables, while supervised-learning approaches can evaluate whether combinations of molecular features improve discrimination or risk stratification. Multi-Omics Factor Analysis (MOFA), DIABLO, and related latent-variable methods provide examples of computational frameworks for integrative multi-omics analysis; however, their application does not overcome limitations arising from inadequate sample size, poor data quality, or insufficient validation [50].

The kidney EV–AI studies identified in this review illustrate both the potential and the current limitations of this strategy. Urinary EV RNA studies have applied machine-learning methods to identify and prioritize candidate molecular signatures in diabetic kidney disease and lupus nephritis [41,42], whereas urinary EV metabolomics and serum EV metabolomics studies have used feature-selection and classification approaches to identify molecular combinations associated with renal involvement [43,45]. Additional urinary EV transcriptomic and EV-associated computational studies further broaden the biomarker-discovery landscape [47,48]. However, substantial heterogeneity in study populations, EV sources, pre-analytical and isolation procedures, omics platforms, feature-selection methods, and validation strategies currently limits direct comparison between studies and precludes meaningful quantitative pooling.

Apparent improvements in predictive performance should therefore be interpreted cautiously, particularly when feature selection and model optimization are performed in small datasets. If data preprocessing, feature selection, hyperparameter tuning, and performance estimation are not appropriately separated within the validation framework, information leakage and overfitting may produce optimistically biased estimates. AI-derived feature importance should also not be interpreted as evidence of biological causality. Influential EV-associated features require confirmation in independent cohorts using standardized EV-processing workflows, together with orthogonal analytical validation and, where feasible, functional investigation.

The translational objective is therefore not simply to maximize AUROC. A clinically useful EV biomarker panel should demonstrate analytical reproducibility, external validity, adequate calibration, clinically meaningful operating characteristics, and incremental value beyond established measures such as eGFR and albuminuria. Prospective multicentre studies will ultimately be required to determine whether AI-selected EV signatures improve clinical decision-making and patient stratification rather than merely distinguish cases from controls in retrospective datasets.

#### 3.4.2. AI-Powered Risk Stratification and Progression Modeling

Machine-learning models incorporating urinary or EV-associated biomarkers have been investigated for CKD risk stratification and progression modeling. As a contextual non-EV comparator, Bienaimé et al. primarily conducted an assay-validation study, with an exploratory prediction analysis using LASSO logistic regression on a panel of urinary biomarkers, including CCL2, EGF, KIM-1, NGAL, and TGF-α, in the NephroTest cohort (*n* = 229; 68 rapid progressors). The five-biomarker model showed greater discrimination for rapid measured-GFR decline than the Kidney Failure Risk Equation alone, with a mean AUC of 0.722 versus 0.682 [25]. However, model performance was evaluated using repeated stratified two-fold cross-validation rather than an independent external validation cohort. This distinction is important because internally validated performance estimates may not reflect model performance in independent populations.

The nine studies included in the PRISMA evidence set are heterogeneous and should not be interpreted as nine equivalent clinical EV–AI prediction studies. Four provide the clearest direct or exploratory kidney EV–AI evidence. Burrello et al. applied supervised learning to serum and urinary EV surface markers in kidney transplant recipients [40]. Zhao et al. developed a machine-learning diagnostic model for type 2 diabetic nephropathy using urinary EV RNA biomarkers, reporting AUCs of 0.877 in the validation cohort and 0.824 in an independent test cohort [41]. In lupus nephritis, urinary EV RNA candidates were prioritized using three machine-learning algorithms and subsequently evaluated in an independent 143-sample validation cohort [42]. A separate lupus-nephritis uEV metabolomics study used random-forest-based feature prioritization and reported AUCs of 0.912, 0.906, and 0.897 for three candidate metabolites [43].

The remaining retained studies broaden the evidence base but require more cautious interpretation. Wang et al. combined serum exosome metabolomics with random forest, LASSO, and support vector machine-recursive feature elimination (SVM-RFE) in systemic lupus erythematosus, reporting a combined AUC of 0.931 for distinguishing lupus nephritis from non-renal SLE [45]. Song et al. compared six machine-learning algorithms in a DKD case–control study, with XGBoost showing the highest reported performance (ROC AUC, 0.877; accuracy, 0.825). However, because the urinary-exosome substudy linked GSK3β-related measures to kidney-disease phenotypes rather than providing a purely EV-derived classifier, this study is best regarded as supporting evidence [46]. Zhao et al. combined whole-transcriptome urinary EV profiling with WGCNA, ceRNA-network analysis, and machine-learning methods in a small T2DN cohort, identifying six candidate RNAs and providing exploratory evidence for EV-RNA biomarker discovery [47]. Yang et al. integrated tissue transcriptomics, Mendelian randomization, XGBoost, and random-forest feature selection to identify EV-associated DKD biomarkers; because the predictors were derived primarily from tissue datasets rather than a direct clinical EV assay, this study provides supporting computational evidence [48]. Bruschi et al. applied nonlinear SVM, partial least-squares discriminant analysis (PLS-DA), and network analysis to mesothelial exosome proteomics from pediatric peritoneal-dialysis effluent. However, the very small FSGS and non-FSGS groups make the reported complete separation exploratory and at high risk of overfitting [49].

Collectively, these nine studies support the feasibility of integrating AI/ML methods with EV-related data across kidney-disease settings. However, substantial differences in study populations, EV sources, analytical platforms, outcomes, sample sizes, and validation strategies preclude their interpretation as a uniform clinical evidence base.

The Klinrisk model provides a useful non-EV comparator for the level of validation expected of a clinically transportable prediction model. It was developed in 77,196 participants from Manitoba and externally validated in 107,097 participants from Alberta using a 22-variable random-forest model. The reported AUCs were 0.88 at two years and 0.84 at five years in the development cohort, compared with 0.87 and 0.84, respectively, in the external validation cohort, with calibration also assessed externally [38]. Although Klinrisk is not an EV-specific model, it illustrates the scale and independent validation that would strengthen claims of transportability for future EV–AI signatures. The distinction between internally validated and externally validated predictive performance is illustrated in Figure 3.

#### 3.4.3. Structured PROBAST Risk of Bias and Applicability Assessment

A structured PROBAST-informed appraisal was conducted using the primary publications rather than secondary reviews. The original PROBAST framework was applied across its four domains: participants, predictors, outcome, and analysis [7]. Domain-level judgments use the standard PROBAST categories Low, High, or Unclear; hybrid categories such as “low–moderate” or “moderate–high” were not used. Because several retained reports were exploratory biomarker-discovery studies rather than formal clinical prediction-model development studies, the appraisal is explicitly described as PROBAST-informed and is interpreted according to each study’s design. All nine retained reports with predictive, classification, or ML-based biomarker analyses are included, together with three non-EV comparator studies used as methodological benchmarks. The 2025 PROBAST + AI update was used as a methodological cross-check [8]. External validation was recorded separately and was not, by itself, used to determine risk of bias. The resulting judgments are summarized in Table 4.

Scoring rationale: Bienaimé et al. was treated primarily as an assay-validation study with an exploratory LASSO prediction analysis rather than as a mature clinical prediction-model study [25]. Ferguson et al. provides a methodological comparator with large development and independent external-validation cohorts [38]. Among the retained EV/EV-associated studies, Zhao et al. 2026 included a time-independent test cohort and reported calibration and decision-curve analyses [41], whereas most other studies were limited by small samples, high-dimensional feature selection, absence of independent external validation, or indirect EV ascertainment. Burrello et al. evaluated 616 candidate models in 58 transplant recipients [40]; Wang et al. analyzed 586 serum exosome-derived metabolites in 91 participants [45]; Zhao et al. 2023 analyzed whole-transcriptome uEV data in 24 participants [47]; and Bruschi et al. used high-dimensional exosome proteomics in very small FSGS and non-FSGS groups [49]. These design features informed the domain-level judgments summarized in Table 4 and Table S2.

#### 3.4.4. Beyond AUROC: Predictive Value, Operating Thresholds, and Explainability

AUROC summarizes a model’s ability to discriminate between individuals with and without an outcome across a range of classification thresholds, but it does not by itself define a clinically actionable operating point. Positive and negative predictive values (PPV and NPV) depend on sensitivity, specificity, the selected operating threshold, and outcome prevalence in the target population. Consequently, a model with a high AUROC may still have a low PPV when applied in a low-prevalence screening population. Most predictive models summarized below report AUC or AUROC without a prespecified operating threshold and corresponding PPV and NPV in the intended-use population. Where a validated or published cutoff is available, as in the case of CKD273, the corresponding operating characteristics should be reported directly rather than inferred from AUROC [28].

Model interpretability is an important consideration in the clinical translation of AI-based prediction tools. Explainable AI (XAI) approaches, including SHAP values, attention-based visualization, and saliency mapping, can identify features that contribute to individual or model-level predictions; however, feature importance should not be interpreted as evidence of biological causality. Highly ranked features may reflect genuine biological associations, but they may also capture batch effects, clinical confounding, or artefacts introduced during EV collection, isolation, processing, or measurement. Clinically credible interpretation of XAI outputs therefore requires reproducibility across independent datasets and standardized laboratory workflows, integration with established biological knowledge, and, where appropriate, experimental and prospective clinical validation.

#### 3.4.5. Digital Pathology and Computational Renal Histology

AI-assisted renal histopathology is relevant to this review despite not being directly EV-based, because tissue morphology can provide spatial and phenotypic context for molecular biomarker findings. Computational pathology methods can quantify glomerular, tubular, interstitial, and vascular features from digitized kidney-biopsy images and have been investigated for automated lesion detection, classification, and grading in kidney disease [52]. In principle, these quantitative histological features could be integrated with urinary or circulating EV profiles to determine whether EV-derived molecular signatures correspond to specific patterns of tissue injury.

A multimodal framework integrating EV-derived molecular data, routine clinical variables, and computational histopathology may be particularly relevant to heterogeneous diseases such as diabetic kidney disease and lupus nephritis, in which histological features provide important information about disease phenotype and severity. However, such integration remains a future research direction rather than an established AIVEV workflow. Demonstrating incremental clinical value would require paired biopsy and EV datasets, standardized EV-processing procedures, harmonized histopathological annotation, independent external validation, and evidence that multimodal models improve clinically relevant prediction or decision-making beyond models based on histopathology, EV biomarkers, or established clinical variables alone.

### 3.5. AIVEVs for CKD Treatment: Computational Design of Therapeutic EVs

Scope note: Section 3.5 and Section 3.6 are contextual narrative extensions informed by targeted literature on EV engineering, therapeutic development, manufacturing, and PBPK modeling. They are not findings derived from the nine-study PRISMA evidence set, which consisted of diagnostic, prognostic, biomarker-discovery, and EV-associated computational studies.

#### 3.5.1. The Therapeutic EV Engineering Pipeline

Developing EVs as therapeutic candidates for CKD requires coordinated optimization of cargo selection, renal targeting, biodistribution, loading efficiency, stability, safety, and scalable manufacturing. AI-based approaches may help prioritize candidate designs across these stages; however, computational optimization must remain constrained by biological feasibility, pharmacological considerations, and the requirements of the intended manufacturing process [15,51].

An integrated therapeutic-development pipeline should therefore link biological target identification with cargo selection, EV surface engineering, pharmacokinetic/pharmacodynamic (PK/PD) modeling, experimental validation, and manufacturing considerations rather than treating these components as independent steps. Candidate EV designs should undergo sequential evaluation of identity, purity, potency, biodistribution, safety, batch-to-batch reproducibility, and release criteria. Such constraints are particularly important for generative or optimization-based computational approaches, because a design predicted to have favorable biological properties may nevertheless be unsuitable for translation if it requires impractical cargo stoichiometry, unstable membrane composition, low-yield production, or non-scalable isolation or loading procedures. This constrained and iterative framework is summarized in Figure 4 and Table 5.

#### 3.5.2. Kidney-Targeted EV Surface Engineering

Achieving therapeutically relevant renal delivery while minimizing systemic exposure remains a major challenge in the development of EV-based therapies for kidney disease. Several targeting strategies have been investigated experimentally and could, in principle, provide design constraints or candidate features for future AI-assisted optimization.

KIM-1 targeting: Kidney injury molecule-1 (KIM-1/HAVCR1) is upregulated in injured tubular epithelial cells and therefore represents a potential target for site-directed delivery. In a preclinical AKI study, red blood cell-derived EVs functionalized with the KIM-1-binding LTH peptide accumulated at injured renal tubules and were used to deliver therapeutic siRNAs [53]. This is an AKI proof-of-concept rather than CKD clinical evidence. In future applications, computational or AI-assisted peptide-design approaches could potentially be used to optimize properties such as binding affinity, selectivity, stability, and immunogenicity; however, such optimization would require experimental validation.

RGD peptide engineering: The arginine–glycine–aspartic acid (RGD) motif interacts with integrins expressed by several cell types involved in tissue injury and fibrosis. RGD-containing supramolecular nanofibers have been investigated as a strategy to enhance MSC-EV-mediated delivery of let-7a-5p, with reported effects on apoptosis and autophagy involving CASP3 and RragD [14]. Generative or optimization-based computational methods could potentially be used to explore RGD variants with favorable targeting and pharmacological properties, but superiority over existing constructs would need to be demonstrated experimentally.

Neutrophil membrane camouflage: Engineering EV-based delivery systems with neutrophil-derived membrane components represents another strategy for exploiting the inflammatory homing properties of neutrophils. Such hybrid vesicular systems have shown preferential localization to injured renal tissue and therapeutic effects in preclinical models of acute kidney injury [14]. Computational analysis of neutrophil surface-protein profiles could potentially assist in identifying membrane components relevant to renal inflammatory targeting, although CKD-specific efficacy remains to be established.

Glycoengineering: Glycoengineering of stem-cell-derived EVs represents an additional targeting strategy in which surface carbohydrate structures are modified to influence interactions with recipient cells. Glycoengineered EVs have been investigated for targeted delivery in preclinical models of acute kidney injury [54]. Machine-learning approaches could potentially assist in modeling glycan–receptor interactions and prioritizing candidate surface modifications; however, this application remains an emerging computational strategy requiring experimental and translational validation.

#### 3.5.3. Generative Models for Virtual EV Design

The concept of a “virtual EV”—a computational representation that could be designed, evaluated, and iteratively refined in silico before experimental testing—represents a potential future direction for therapeutic EV development. Generative approaches, including variational autoencoders (VAEs) and generative adversarial networks (GANs), can learn latent representations from sufficiently large and well-characterized datasets and generate candidate configurations for subsequent evaluation. However, their application to EV therapeutic design remains an emerging concept rather than an established or clinically validated workflow [34].

A future computational platform for EV design could integrate multiple complementary approaches, including protein-structure prediction for evaluating candidate cargo and surface proteins; molecular-dynamics simulations for investigating membrane and molecular interactions; reinforcement-learning or multi-objective optimization methods for balancing properties such as targeting, cargo loading, stability, and manufacturability; and digital-twin frameworks for exploring patient-specific pharmacological responses. These approaches should be regarded as components of a proposed computational framework rather than as validated elements of current AIVEV development.

Computational design could, in principle, reduce experimental iteration by prioritizing candidate cargos, targeting ligands, surface modifications, and process conditions before laboratory testing. However, current EV-specific evidence does not establish a reproducible quantitative reduction in development time or experimental burden. Such potential efficiencies should therefore be considered hypotheses requiring prospective experimental evaluation rather than established performance claims.

Importantly, the translational value of an in silico EV design ultimately depends on its experimental and manufacturing feasibility. Generative-design pipelines optimized solely for predicted biological properties, such as targeting, cargo loading, or stability, could propose constructs that cannot be produced with the yield, purity, stability, reproducibility, or cost required for clinical development. A translationally relevant AIVEV design framework should therefore incorporate manufacturability constraints at an early stage, including achievable producer-cell yields, scalability of EV isolation and cargo-loading procedures, batch-to-batch reproducibility, requirements for Chemistry, Manufacturing, and Controls (CMC), and anticipated manufacturing complexity and cost. These considerations should function as design constraints or feasibility criteria rather than being deferred until after a candidate design has been selected.

### 3.6. Physiologically Based Pharmacokinetic Modeling for Renal EV Delivery

Physiologically based pharmacokinetic (PBPK) modeling provides a mechanistic framework for describing and predicting the biodistribution and disposition of administered EVs across tissues and organs. By integrating physiological parameters with EV-specific properties and experimental biodistribution data, PBPK models may help characterize renal exposure, systemic distribution, and clearance and support the translation of preclinical findings across species and dosing regimens [55].

#### 3.6.1. PBPK Model Structure for EVs

A whole-body PBPK model for EVs may include compartments representing major organs involved in EV distribution and clearance, including the kidney, liver, spleen, lungs, heart, and brain, interconnected through the systemic circulation. Relevant model parameters may include EV size distribution and surface properties, including zeta potential; organ blood-flow rates and vascular permeability; and tissue-specific uptake and clearance processes, particularly those involving cells of the mononuclear phagocyte system [55].

Glomerular filtration is expected to contribute only minimally to the clearance of intact EVs because most EV populations exceed the effective size range for passage across the glomerular filtration barrier. Nevertheless, EV biodistribution and clearance cannot be predicted from particle size alone and may also be influenced by surface composition and charge, protein-corona formation, interactions with endothelial and immune cells, tissue permeability, and disease-associated changes in organ physiology.

For EVs specifically engineered for renal delivery, additional processes may need to be considered, including transport across the renal microvasculature, cellular uptake by tubular and other renal cell populations, intracellular processing and cargo release, and, where relevant, trafficking within the urinary compartment. These processes should be distinguished from the physiological release of endogenous urinary EVs, because the presence of EVs in urine does not necessarily indicate renal excretion of intact systemically administered EVs.

During early-stage model development, a reduced PBPK representation comprising systemic circulation, target tissue, and non-target tissues may provide a useful framework for hypothesis generation and parameter exploration. Such models can incorporate simplified clearance kinetics and pharmacodynamic concentration–effect relationships where supported by experimental data. However, the appropriate model structure and degree of complexity should be determined by the biological question, route of administration, available biodistribution data, and intended application rather than assumed a priori [55].

#### 3.6.2. AI-Enhanced PBPK Parameterization

Machine-learning approaches may potentially address several challenges in the parameterization and application of PBPK models for EVs. For example, probabilistic and Bayesian methods could assist in estimating uncertain model parameters from sparse experimental data, while machine-learning approaches may help characterize nonlinear relationships relevant to interspecies scaling. Gaussian-process and other probabilistic models could also be used to represent interindividual variability and quantify uncertainty in EV pharmacokinetic parameters. In principle, data-driven approaches could further assist in modeling disease-associated physiological changes, such as altered renal perfusion, vascular permeability, inflammatory status, and tissue uptake, that may influence EV biodistribution in CKD. However, these applications remain largely methodological possibilities rather than established EV-specific modeling strategies.

At present, AI-enhanced PBPK approaches should not be regarded as validated tools for CKD-specific EV dose selection. EV biodistribution datasets remain limited, assay-dependent, and heterogeneous across species, EV sources, formulations, and experimental conditions. Under these circumstances, highly flexible machine-learning models may propagate or amplify uncertainty rather than necessarily reduce it. AI-enhanced PBPK models should therefore retain physiologically plausible mechanistic constraints, explicitly quantify parameter uncertainty, undergo sensitivity and identifiability analyses, and be evaluated against independent biodistribution and pharmacokinetic data before being used to support dose selection or interspecies translation [55].

## 4. Discussion

### 4.1. Clinical Translation: Challenges and Regulatory Considerations

#### 4.1.1. The Translation Funnel

Despite encouraging preclinical findings, translating EV-based approaches into clinically validated CKD therapies remains challenging. The preclinical evidence base substantially exceeds the available clinical evidence, and no EV-based therapy has yet been approved specifically for CKD. One registered clinical study, VE-IRC, is evaluating EVs as biomarkers in chronic renal failure [56]; however, this is a diagnostic biomarker study rather than a therapeutic EV trial. The translational gap reflects interconnected challenges, including EV heterogeneity, manufacturing and batch-to-batch consistency, dose selection, biodistribution, safety, regulatory standardization, and prospective demonstration of clinical efficacy. Figure 5 summarizes these barriers and the transition from preclinical development to clinical implementation, while Table 6 summarizes key translational challenges and potential AI-driven solutions.

#### 4.1.2. Regulatory Pathways

The regulatory framework for EV-based therapeutics continues to evolve. Although the International Society for Extracellular Vesicles (ISEV) is not a regulatory authority, its MISEV2023 guidelines provide important scientific recommendations for EV research, including transparent reporting of EV separation and characterization procedures, the appropriate use of complementary characterization methods, and rigorous documentation of experimental conditions [30]. Such standardization is important for generating reproducible evidence that can support subsequent therapeutic development and regulatory evaluation.

When AI/ML components contribute to diagnosis, prediction, treatment selection, or clinical decision support, additional regulatory considerations may apply depending on the intended use and regulatory classification of the software component. Relevant considerations include algorithm transparency and interpretability, the representativeness of training and validation datasets, identification and mitigation of algorithmic bias, management of model updates and performance drift, appropriate human oversight, and clear definition of how model outputs are incorporated into clinical decision-making. For software components that meet the applicable definition of Software as a Medical Device (SaMD), these considerations may form part of the broader regulatory evaluation.

Explainable AI (XAI) methods, including SHAP-based feature-attribution approaches and attention visualization, may support model interpretation and facilitate assessment of model behavior [34]. However, explainability alone does not establish model validity, biological plausibility, safety, or clinical utility. As discussed in Section 3.4.4, XAI outputs should therefore be evaluated for reproducibility and interpreted alongside independent biological evidence and clinical validation rather than being regarded as intrinsically trustworthy.

### 4.2. Limitations

This scoping review has limitations related to both the review process and the underlying evidence base. The primary structured database search was restricted to PubMed/MEDLINE and supplemented by backward citation tracking and focused searches of the computational literature; comprehensive searches of Scopus, Web of Science, Embase, and IEEE Xplore were not performed. Relevant engineering or computational studies indexed exclusively outside PubMed/MEDLINE may therefore have been missed. The review was also not prospectively registered, and no protocol was deposited in a public registry before the review was conducted. These limitations should be considered when interpreting the comprehensiveness and reproducibility of the resulting evidence map.

The underlying literature is also heterogeneous. The nine retained reports comprise direct kidney EV–AI studies, EV-associated machine-learning studies, and supporting computational or omics investigations rather than a uniform set of clinical prediction studies. Cohorts are often small, EV pre-analytical and analytical workflows vary across studies, independent external validation is uncommon, and calibration, prespecified operating thresholds, and prospective clinical utility are incompletely reported. The PROBAST assessment was therefore used to structure the critical appraisal of predictive-model evidence rather than to imply that all retained studies represent equivalent prediction models. This clinical and methodological heterogeneity precluded quantitative meta-analysis and warrants cautious interpretation of the reported performance estimates.

### 4.3. Future Directions

Current evidence supports the feasibility of integrating artificial intelligence with extracellular vesicle-based biomarker discovery and therapeutic design in CKD but does not establish clinical readiness. Direct kidney EV–AI studies remain limited, disease-specific, and heterogeneous, with methodological concerns related to cohort size, pre-analytical variability, predictor dimensionality, validation strategy, calibration, and clinical utility. Methodological precedents from larger non-EV prediction studies demonstrate the importance of external validation and model transportability but should not be interpreted as evidence that EV-derived models provide equivalent or superior predictive performance.

Several emerging computational approaches may contribute to the future development of AIVEV platforms. Multimodal learning could integrate EV-derived molecular profiles with clinical, imaging, histopathological, and longitudinal data, although its incremental value in CKD EV applications requires prospective evaluation. Federated learning may support multicentre model development and validation while allowing patient-level data to remain within participating institutions; however, cross-site heterogeneity, data governance, privacy, and technical interoperability remain important challenges [35,57]. Digital-twin approaches may eventually support individualized simulation and hypothesis generation by integrating longitudinal clinical and biological data; however, current nephrology applications remain research frameworks rather than validated clinical tools [58]. Similarly, AI-assisted optimization of EV engineering and manufacturing may facilitate candidate prioritization and process development [59], but evidence of scalability, reproducibility, safety, and cost-effectiveness remains limited.

Future progress will require larger multicentre cohorts; standardized and transparently reported EV collection, isolation, and characterization workflows; rigorous model development and independent external validation; clinically relevant, prespecified operating thresholds; and prospective evaluation of clinical utility. Therapeutic development will also require explicit consideration of manufacturability, quality control, batch-to-batch reproducibility, biodistribution, safety, and regulatory requirements. Computational approaches, including multimodal learning, federated learning, generative modeling, digital-twin frameworks, and PBPK-informed optimization, may contribute to these goals, but their value in CKD EV applications must ultimately be established through rigorous experimental and clinical validation.

## 5. Conclusions

The convergence of artificial intelligence and extracellular vesicle research offers a promising framework for developing more informative CKD biomarkers and more rationally engineered EV therapeutics; however, the field remains at an early stage of clinical translation. The studies mapped in this scoping review demonstrate feasibility across urinary EV RNA, metabolomic, surface-marker, and related computational applications but do not establish a clinically validated AIVEV diagnostic or therapeutic platform.

AIVEV should therefore be regarded as an emerging research framework rather than an established clinical technology or regulatory category. Progress will depend less on increasing algorithmic complexity than on reproducible EV biology, standardized pre-analytical workflows, adequately powered multicentre cohorts, independent external validation, assessment of calibration and clinically relevant operating thresholds, prospective evaluation of clinical utility, and early consideration of manufacturability, safety, quality control, and regulatory requirements. AI is likely to be most valuable when it prioritizes experimentally testable hypotheses and supports transparent, biologically grounded decision-making. Ultimately, clinical translation will require prospective evidence that AIVEV-based approaches improve clinically meaningful prediction, decision-making, or patient outcomes beyond established CKD assessment and management strategies.

## Figures and Tables

**Figure 2:**
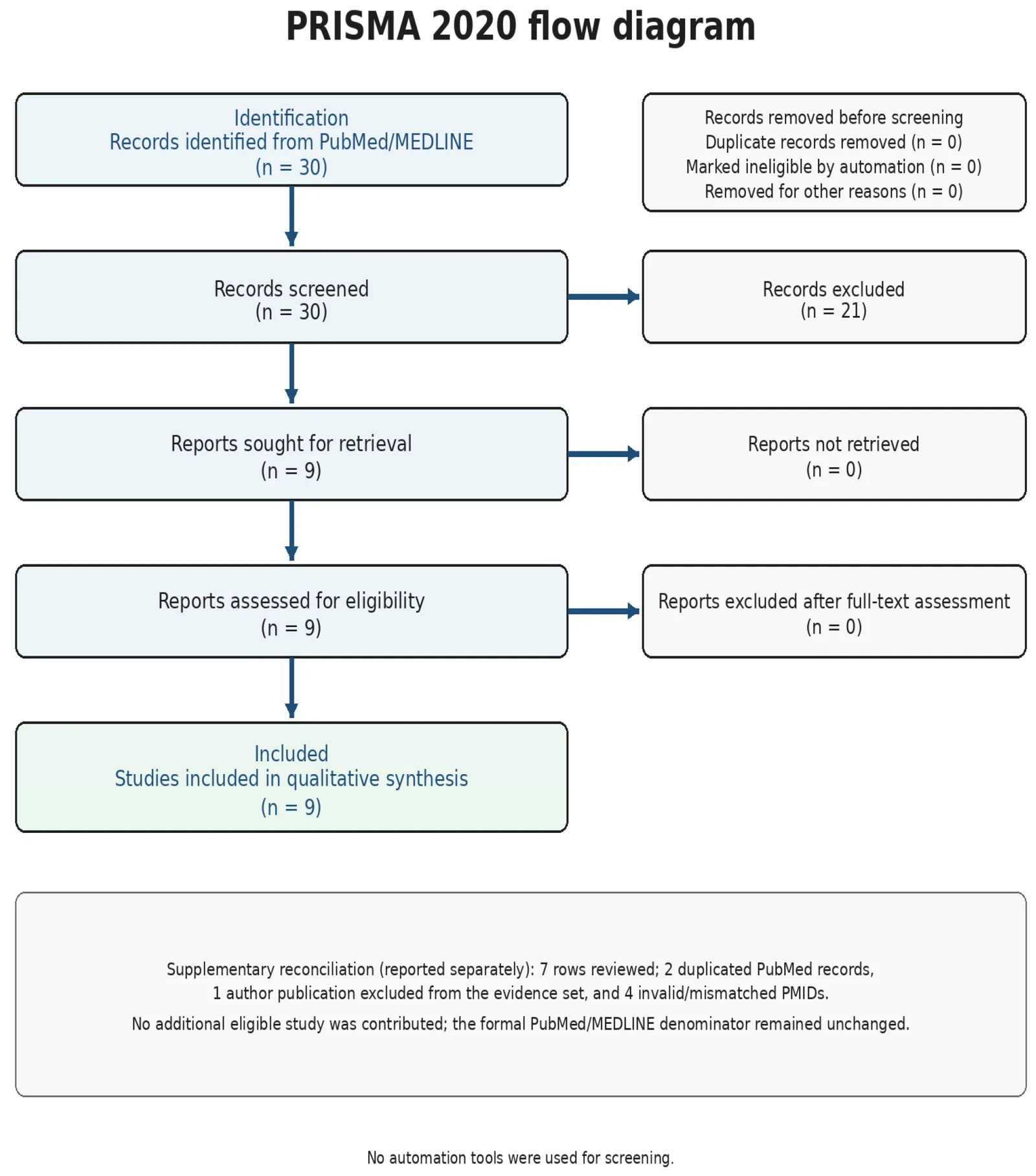
PRISMA 2020 flow diagram for the documented study-selection process [6]. The archived PubMed/MEDLINE export contained 30 records; 21 were excluded at title/abstract screening, nine reports proceeded to full-text assessment, and all nine were retained for qualitative synthesis. The separate seven-row supplementary reconciliation is reported outside the formal PRISMA denominator: two rows duplicated PubMed records, one was an author publication excluded from the evidence set, and four contained invalid or mismatched PMIDs; it contributed no new eligible study. Arrows indicate progression through identification, screening, eligibility assessment, and inclusion; the colored boxes distinguish the main process stages and the final included-study set.

**Figure 3:**
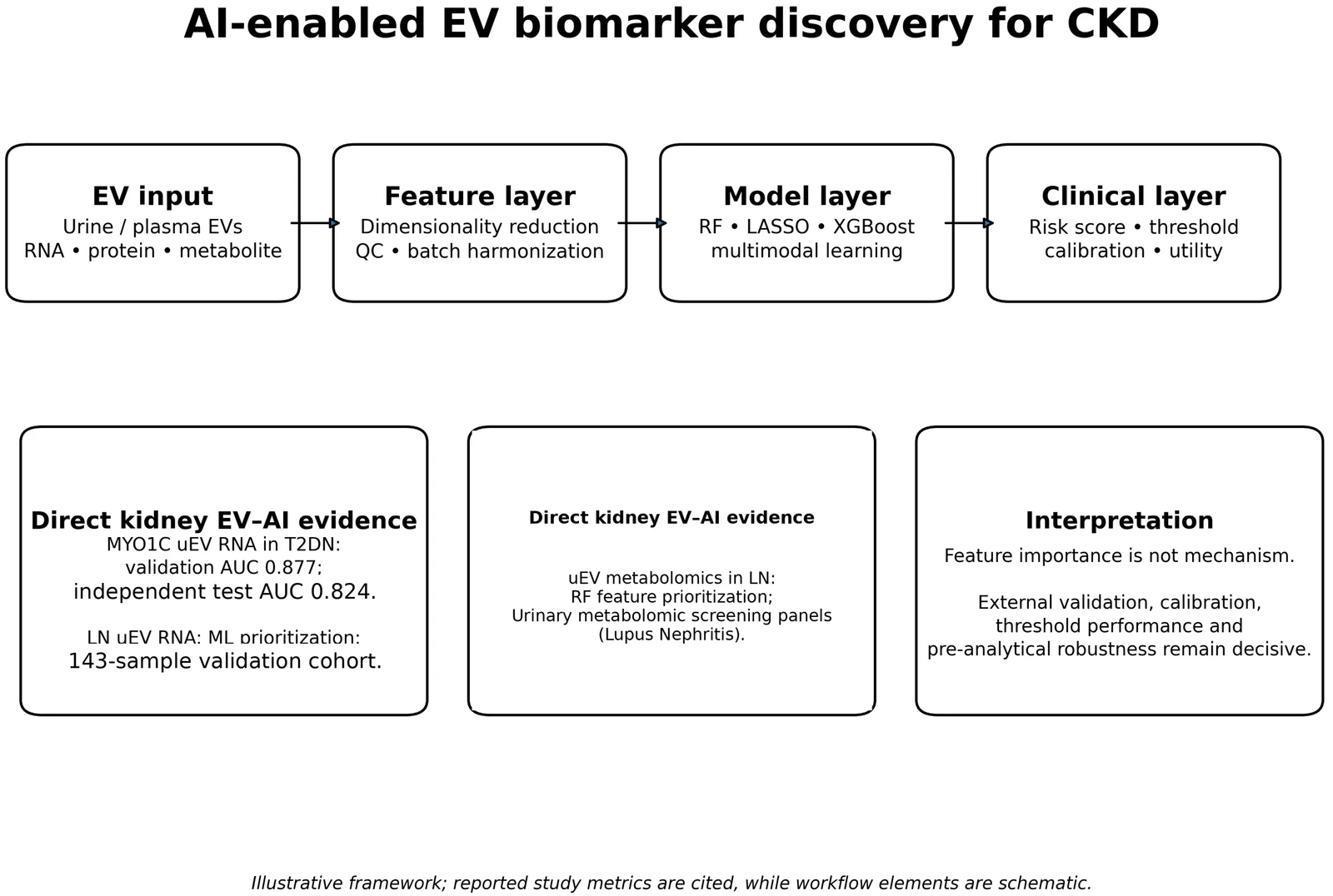
AI-enabled EV biomarker discovery for CKD. The workflow is schematic. Reported study metrics are cited in the text; the architecture and evidence categories are not model outputs from a single dataset.

**Figure 4:**
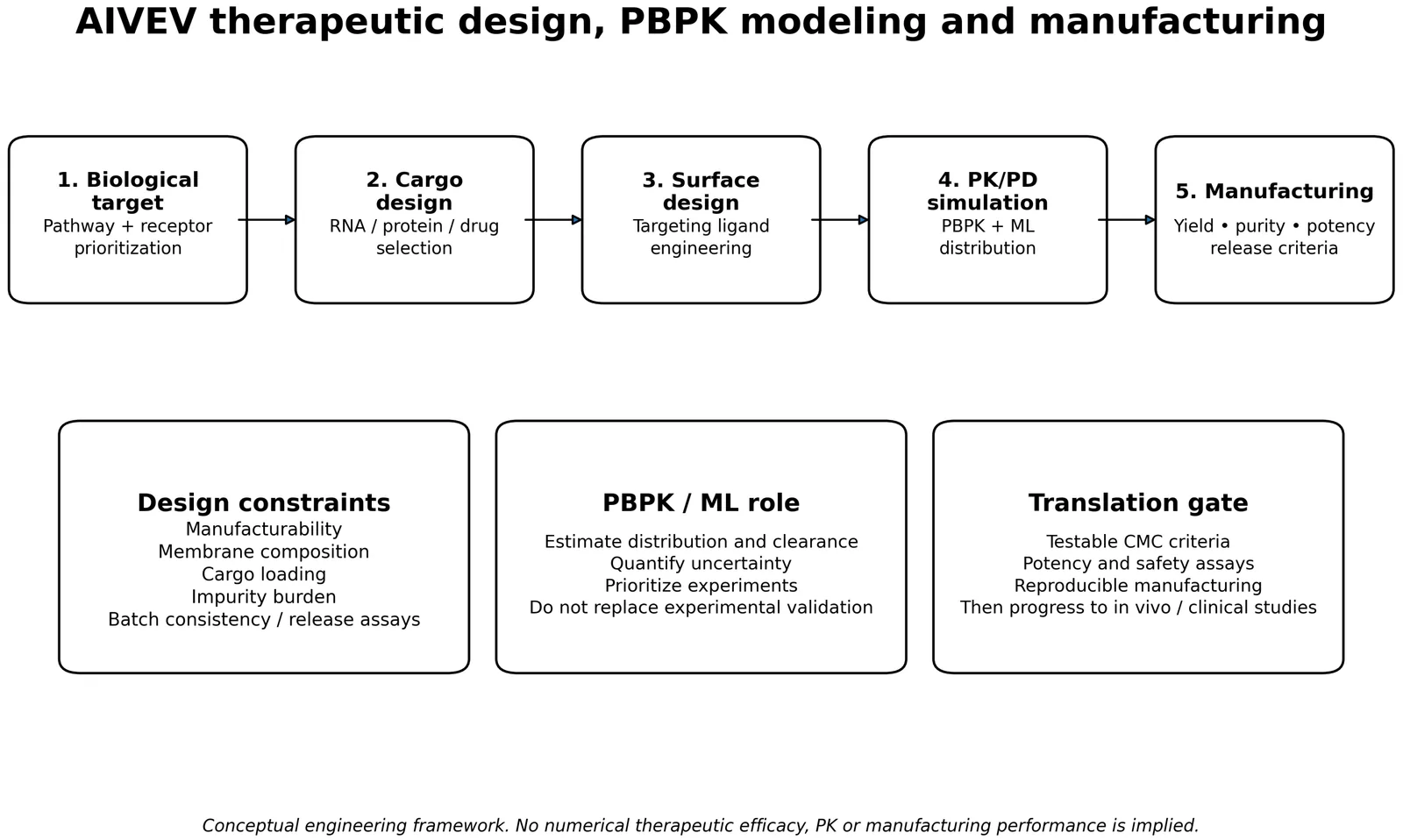
AIVEV therapeutic design, PBPK modeling and manufacturing. The workflow is conceptual and emphasizes manufacturability, quality control, potency and release criteria as design constraints. No numerical therapeutic efficacy or manufacturing performance is implied.

**Figure 5:**
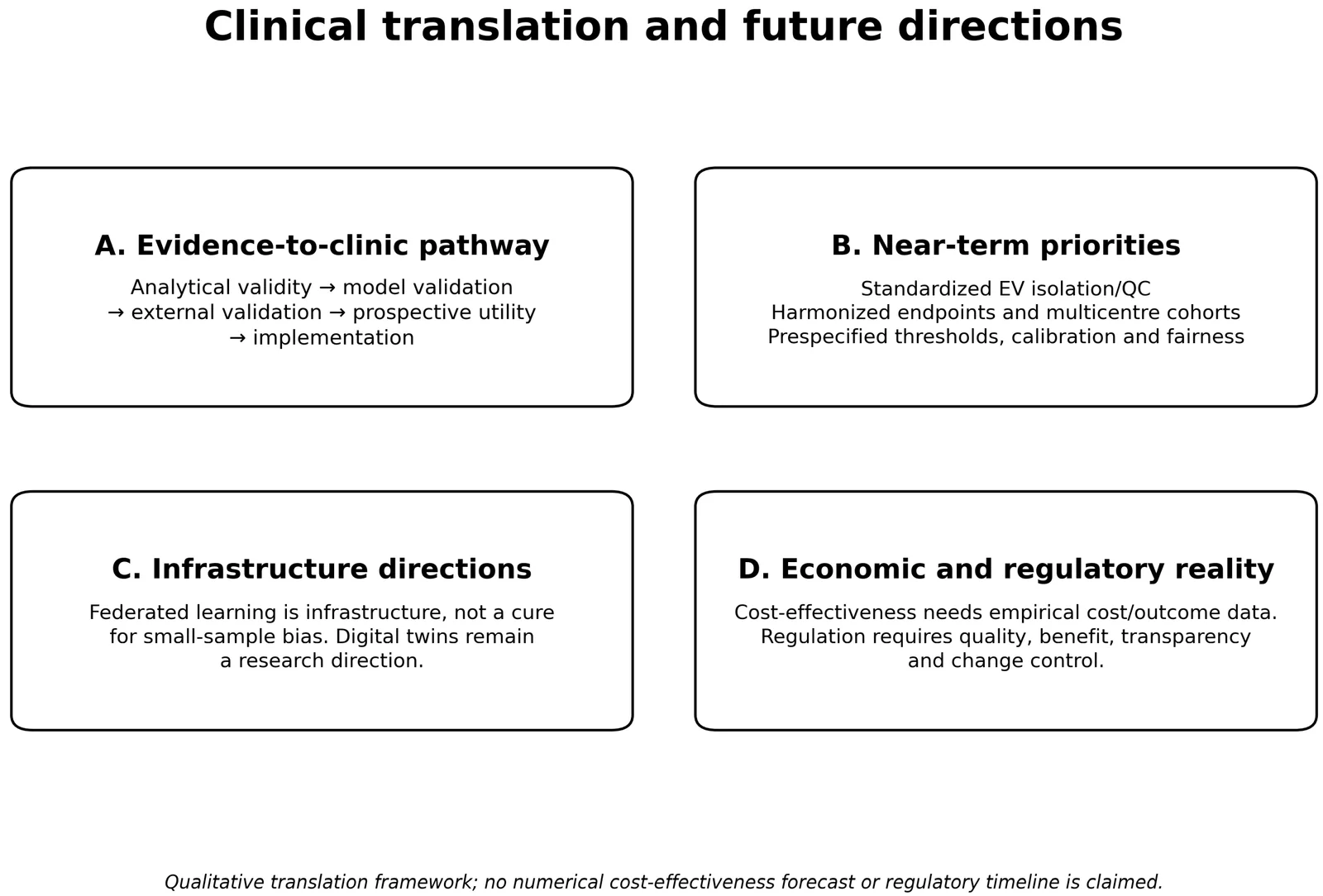
Clinical translation and future directions. The panels summarize translational evidence gates, infrastructure priorities, and regulatory and economic considerations. The figure is qualitative and does not present numerical cost-effectiveness projections or forecasted regulatory timelines.

**Table 1:** Global Epidemiology of Chronic Kidney Disease (2021–2023).

Parameter	Estimate	Source/Year
Global prevalence (adults ≥ 20 y)	788 million (95% UI 743–843)	GBD 2023 [1]
Age-standardized prevalence	14.2% (adults ≥ 20 years)	GBD 2023 [1]
Separate advocacy estimate (all kidney disease/CKD subset)	850 million/843 million	ERA-ISN-ASN, cited in [9]
Annual deaths	1.5 million (approx. one every 20 s)	GBD/Ortiz et al., 2026 [9]
Patients on kidney replacement therapy	4.6 million	GBD 2023 KFRT [10]
Highest regional age-standardized prevalence	North Africa & Middle East (18.0%)	GBD 2023 [1]

**Table 2:** Representative urinary and EV-associated biomarkers relevant to CKD diagnosis and prognosis.

Biomarker	Source/Compartment	Direction in CKD/Fibrosis	Clinical or Pathological Association	Evidence Type	Key Supporting Literature
miR-21	Urinary exosomes/uEVs	Upregulated	Fibrosis severity; inverse correlation with eGFR; elevated in LN chronicity	Direct urinary-EV evidence	Lange et al., 2019 [16]; Solé et al., 2019 [18]
miR-29c	Urinary exosomes/microvesicles	Downregulated	Tubulointerstitial fibrosis degree; predicts chronicity in LN	Direct urinary-EV evidence	Lv et al., 2013 [19]
miR-200b	Urinary exosomes (non-proximal tubule-derived)	Downregulated	Progressive renal fibrosis	Direct urinary-EV evidence	Yu et al., 2018 [20]
CCL2 mRNA	Tubular epithelial cell-derived EVs/uEVs	Upregulated	Interstitial inflammation, proteinuria (IgA nephropathy)	Direct EV/mechanistic evidence	Lv/Feng et al., 2018 [21]
NGAL	Urinary EVs/exosome-like EV fraction; also free urinary NGAL	Context-dependent	Tubular injury; DKD and other renal-injury contexts	Direct urinary-EV evidence plus broader free-urinary biomarker evidence	Ugarte et al., 2022 [22]; Chirackal et al., 2019 [23]; Devarajan 2010 [24]
KIM-1	Urinary (free; not established here as EV cargo)	Upregulated in tubular injury	Tubular injury and CKD progression-risk context	Non-EV urinary biomarker comparator	Bienaimé et al., 2023 [25]
EGF	Urinary (not specifically shown as EV cargo in primary progression studies)	Downregulated	Predicts GFR decline	Urinary biomarker (not EV-specific)	Bienaimé-type urinary panels [25]; Ju et al. [26]
PON1	Circulating EVs/plasma-associated context	Candidate; direction requires direct CKD EV validation	CKD-associated oxidative/cardiovascular-risk context	Indirect/contextual EV biomarker evidence	Garcia et al. [3]
circRNAs	Urinary exosomes	Correlated with fibrosis	Renal fibrosis specificity (exploratory)	Direct urinary-EV evidence (early)	Cao et al., 2022 [27]
CKD273 peptide panel	Urinary peptides (not EV-restricted)	Differential pattern	CKD progression prediction	Non-EV comparator	Schanstra et al., 2015 [28]

**Table 4:** PROBAST-informed risk-of-bias and applicability assessment of the nine retained EV/EV-associated studies and selected non-EV methodological comparators.

Primary Study/Evidence Role	Participants	Predictors	Outcome	Analysis	Overall ROB/Applicability
Bienaimé et al., 2023 [25]—contextual non-EV comparator; primarily an assay-validation study with an exploratory prediction analysis	Low	Low	Low	High	High—repeated internal resampling; no independent external validation.
Ferguson et al., 2022 [38]—contextual non-EV comparator	Low	Low	Low	Low	Low—large development and independent external-validation cohorts; calibration assessed.
Mendapara 2024 [51]—contextual non-EV genomic comparator	Unclear	Low	Low	High	High—high-dimensional feature selection relative to sample size despite independent GEO evaluation.
Burrello et al., 2023 [40]—retained; direct serum/urine EV prediction	Low	Low	Low	High	High—n = 58, 37 EV antigens, 616 candidate models; leave-one-out validation.
Zhao et al., 2026 [41]—retained; direct uEV RNA diagnostic model	Low	Low	Low	Low	Low—time-independent test cohort, calibration and decision-curve analysis reported.
Zhang H et al., 2025 [42]—retained; direct uEV RNA biomarker/ML study	Low	Low	Low	High	High—small discovery cohort with high-dimensional RNA screening; independent biomarker validation.
Zhang N et al., 2026 [43]—retained; direct uEV metabolomics/ML study	Low	Low	Low	High	High—high-dimensional metabolomics and RF/RFECV; no independent external validation.
Wang et al., 2025 [45]—retained; serum exosome metabolomics/ML	Low	Low	Low	High	High—91 participants, 586 metabolites, multiple feature-selection algorithms; no external validation.
Song et al., 2025 [46]—retained; DKD ML study with urinary-exosome substudy	Low	Low	Low	High	High—classifier was not a purely EV-derived model; EV measurements were supporting.
Zhao et al., 2023 [47]—retained; exploratory uEV transcriptomics/ML	Low	Low	Low	High	High—24 participants, high-dimensional transcriptomics; no independent clinical validation.
Yang et al., 2025 [48]—retained; EV-associated computational study, not direct EV assay	Unclear	Low	Low	High	High—public tissue datasets and computational feature selection; limited applicability to direct EV testing.
Bruschi et al., 2021 [49]—retained; mesothelial exosome proteomics/ML	High	Low	Low	High	High—extremely small groups with high-dimensional proteomics; no independent validation.

Note: Table 4 includes all nine reports retained in the PRISMA-ScR evidence set that contained predictive, classification, or ML-based biomarker analyses, together with three non-EV comparator studies used to illustrate methodological standards. PROBAST categories are reported as Low, High, or Unclear.

**Table 5:** AI Applications in Therapeutic EV Engineering for CKD.

Engineering Stage	AI Approach	Application	Potential Design Objective
Target identification	scRNA-seq + pathway mining	Identify dysregulated pathways and receptors in CKD kidneys	Improved candidate prioritization
Cargo selection	NLP on biomedical literature + GNNs	Predict optimal miRNA/protein cargo for pathway modulation	Potentially enhanced therapeutic activity
Surface modification	Molecular docking + RL	Design targeting peptides (e.g., KIM-1, RGD) for renal accumulation	Potentially enhanced renal targeting
Loading optimization	VAEs + experimental design	Predict optimal loading conditions (electroporation, sonication)	Improved cargo-loading efficiency
Stability prediction	Physics-informed neural networks	Model EV membrane dynamics, protein corona formation	Improved stability prediction
PK/PD modeling	PBPK + ML	Predict tissue distribution, clearance, dose–response	Support for dose-selection hypotheses
Safety profiling	Toxicity prediction models	Assess immunogenicity, off-target effects	Improved safety-risk prioritization

**Table 6:** Key Challenges and Potential AI-Driven Solutions for AIVEV Clinical Translation.

Challenge	Traditional Limitation	AI-Driven Solution	Implementation Status
EV isolation standardization	High inter-laboratory variability	ML-based quality control and batch effect correction	Evidence gap; MISEV2023 provides reporting framework, but AI-based harmonization is not established in CKD-EV studies
EV population heterogeneity	Bulk analysis masks rare subpopulations	Deep learning subtype classification; single-vesicle analysis	Evidence mainly outside CKD; CKD application remains exploratory
Limited clinical validation data	Small cohorts, single-center studies	Federated learning across institutions; synthetic data generation	Conceptual/infrastructure direction; federated learning does not solve small-sample bias
Kidney-specific targeting	Low therapeutic index	GNN-based receptor mapping; computational peptide design	Preclinical/computational direction
Manufacturing scalability	Batch-to-batch inconsistency	Process analytical technology (PAT) with real-time ML monitoring	Preclinical/industrial engineering direction; CKD-EV clinical adoption not established
Regulatory approval pathways	Unclear classification (biologic vs. drug vs. device)	Explainable AI for regulatory submission; digital evidence packages	No established AIVEV regulatory pathway; evidence requirements remain product-specific
Cost-effectiveness	High production costs	AI-optimized bioprocess design; predictive maintenance	Empirical health-economic evidence for CKD AIVEV not established

## Data Availability

No new primary datasets were generated for this review. The review is based on published sources; screening, study-characterization, and risk-of-bias details are provided in the Supplementary Materials.

## Supplementary Materials

The following supporting information can be downloaded at: https://www.mdpi.com/article/10.3390/medicina62101878/s1. Table S1: PRISMA-ScR reporting checklist; Table S2: Domain-level PROBAST-informed risk-of-bias judgments for all nine retained EV/EV-associated reports and selected non-EV methodological comparators; Table S3: Studies retained for qualitative synthesis (n = 9); Table S4: Screening reconciliation summary. The reporting and appraisal supplementary files cite references [6,7,8], all of which are included in the main manuscript reference list; File S1: PRISMA 2020 Checklist—Completed; File S2: PRISMA 2020 Line-by-Line Audit.

## Abbreviations

AI	Artificial Intelligence
AIVEV(s)	Artificial Intelligence–Virtual Extracellular Vesicle(s)
AKI	Acute Kidney Injury
AUC	Area Under the Curve
AUROC	Area Under the Receiver Operating Characteristic curve
CCL2	C-C motif Chemokine Ligand 2
circRNA	Circular RNA
CKD	Chronic Kidney Disease
CKD273	A 273-peptide urinary classifier panel for CKD progression prediction
CNN(s)	Convolutional Neural Network(s)
cryo-EM	Cryogenic Electron Microscopy
DALYs	Disability-Adjusted Life Years
DCR-IEVN	DNA Cascade Reaction-triggered Individual EV Nanoencapsulation
DIABLO	Data Integration Analysis for Biomarker discovery using Latent variable approaches for Omics studies
DKD	Diabetic Kidney Disease
EGF	Epidermal Growth Factor
eGFR	estimated Glomerular Filtration Rate
EMR	Electronic Medical Record
ERA	European Renal Association
EV(s)	Extracellular Vesicle(s)
GBD	Global Burden of Disease (Study)
GFR	Glomerular Filtration Rate
GNN(s)	Graph Neural Network(s)
HAVCR1	Hepatitis A Virus Cellular Receptor 1 (gene encoding KIM-1)
ISEV	International Society for Extracellular Vesicles
KDIGO	Kidney Disease: Improving Global Outcomes
KIM-1	Kidney Injury Molecule-1
KFRT	Kidney Failure with Replacement Therapy
LASSO	Least Absolute Shrinkage and Selection Operator
LTH peptide	KIM-1-targeting peptide sequence used for EV surface conjugation
MISEV2023	Minimal Information for Studies of Extracellular Vesicles (2023 guidelines)
ML	Machine Learning
MOFA	Multi-Omics Factor Analysis
mRNA	messenger RNA
MSC(s)	Mesenchymal Stromal Cell(s)
MSC-EV(s)	Mesenchymal Stromal Cell-derived Extracellular Vesicle(s)
MVBs	Multivesicular Bodies
NGAL	Neutrophil Gelatinase-Associated Lipocalin
NLP	Natural Language Processing
NTA	Nanoparticle Tracking Analysis
PAS	Periodic Acid–Schiff (stain)
PAT	Process Analytical Technology
PBPK	Physiologically Based Pharmacokinetic
PD	Pharmacodynamic
PK	Pharmacokinetic
PON1	Paraoxonase 1
PPV/NPV	Positive/Negative Predictive Value
PRISMA-ScR	Preferred Reporting Items for Systematic reviews and Meta-Analyses extension for Scoping Reviews
PROBAST	Prediction model Risk Of Bias ASsessment Tool
RF	Random Forest
RGD	Arginine-Glycine-Aspartic acid (peptide motif)
RL	Reinforcement Learning
RNase	Ribonuclease
SaMD	Software as a Medical Device
scRNA-seq	single-cell RNA sequencing
SGLT2	Sodium-Glucose Cotransporter-2
SHAP	SHapley Additive exPlanations
SP-IRIS	Single-Particle Interferometric Reflectance Imaging Sensor
SVM	Support Vector Machine
TEM	Transmission Electron Microscopy
TGF-α	Transforming Growth Factor alpha
TGF-β1	Transforming Growth Factor beta 1
TIRF	Total Internal Reflection Fluorescence
TNFR1/TNFR2	Tumor Necrosis Factor Receptor 1/2
UACR	Urine Albumin-to-Creatinine Ratio
uEVs	urinary Extracellular Vesicles
UMAP	Uniform Manifold Approximation and Projection
VAE(s)	Variational Autoencoder(s)
WHO	World Health Organization
XAI	Explainable Artificial Intelligence
XGBoost	eXtreme Gradient Boosting
YAP	Yes-Associated Protein
