# Supplementary material for: Artificial Intelligence-Guided Extracellular Vesicle Platforms for Diagnosis and Treatment of Chronic Kidney Disease: A Scoping Review and Future Directions
Source: Medicina (Kaunas). 2026 Sep 28;62(10):1878. doi: 10.3390/medicina62101878 (PMC13706948; doi:10.3390/medicina62101878)
Supplement: Supplementary file 1 [file medicina-62-01878-s001.zip › File S1 PRISMA 2020 Checklist — Completed.pdf]

PRISMA 2020 Checklist — Completed

Artificial Intelligence-Guided Extracellular Vesicle Platforms for Diagnosis and Treatment of Chronic Kidney Disease: A Scoping Review and Future Directions

Manuscript ID: medicina-4534164

Reporting note. This is a scoping review. PRISMA-ScR is the design-specific reporting framework, and this completed PRISMA 2020 checklist provides an additional reporting cross-check. Items not performed, not applicable, or not reconstructable from the archived review materials are stated explicitly rather than inferred.

**Section**	**Item**	**PRISMA 2020 checklist item**	**Location / status in this review**
TITLE	**1**	Identify the report as a systematic review.	Title identifies the article as a scoping review; design-specific terminology retained.
ABSTRACT	**2**	See the PRISMA 2020 for Abstracts checklist.	Abstract: structured design, PubMed/MEDLINE source and 7 September 2026 search date, eligibility focus, non-registration, nine-study evidence set, principal limitations and conclusions are reported.
INTRODUCTION	**3**	Describe the rationale for the review in the context of existing knowledge.	Introduction.
INTRODUCTION	**4**	Provide an explicit statement of the objective(s) or question(s) the review addresses.	Introduction; Materials and Methods — Review framework and question.
METHODS	**5**	Specify inclusion and exclusion criteria and how studies were grouped for syntheses.	Materials and Methods — Eligibility criteria; Study selection; Synthesis approach.
METHODS	**6**	Specify all databases, registers, websites, organisations, reference lists and other sources searched or consulted, and the date each source was last searched.	Materials and Methods — formal evidence source: PubMed/MEDLINE, searched 7 September 2026. Contextual literature identification is explicitly non-systematic and excluded from the PRISMA denominator.
METHODS	**7**	Present the full search strategies for all databases, registers and websites, including filters and limits.	Materials and Methods — full PubMed/MEDLINE search string; no lower publication-date restriction.

METHODS	**8**	Specify methods used to decide whether a study met inclusion criteria, including how many reviewers screened and whether independently; report automation tools if used.	Materials and Methods — Study selection; Screening/data extraction statement. Screening stages and no automation are reported. Archived materials do not permit reliable reconstruction of the number/independence of screeners; this limitation is explicitly disclosed.
METHODS	**9**	Specify methods used to collect data from reports, including how many reviewers collected data and whether independently; describe confirmation/automation where applicable.	Materials and Methods — Screening/data extraction statement. Extraction domains and no automated extraction are reported. Archived materials do not permit reliable reconstruction of the number/independence of extractors; this limitation is explicitly disclosed.
METHODS	**10a**	List and define all outcomes for which data were sought and whether all compatible results were sought.	Materials and Methods — screening/data-extraction domains; formal evidence mapping focused on diagnosis, prognosis, biomarker discovery and related computational analysis.
METHODS	**10b**	List and define all other variables for which data were sought and describe assumptions about missing or unclear information.	Materials and Methods — population, EV source/pre-analytics, predictor modality, AI method, outcome, validation, performance, calibration/clinical utility and translational limitations.
METHODS	**11**	Specify methods used to assess risk of bias in included studies, including tools, reviewers and automation if applicable.	Materials and Methods — PROBAST-informed assessment; Table 4; Supplementary Table S2. The appraisal method is reported, but archived materials do not permit reliable reconstruction of the number/independence of appraisers; this is explicitly disclosed.
METHODS	**12**	Specify effect measures	Not applicable as a uniform

		used for each outcome.	synthesis measure: heterogeneous diagnostic/biomarker studies were mapped narratively; study-reported discrimination/performance metrics are presented where available.
METHODS	**13a**	Describe processes used to decide which studies were eligible for each synthesis.	Materials and Methods — Study selection and Synthesis approach; contextual engineering/future-direction literature explicitly separated from the nine-study formal evidence set.
METHODS	**13b**	Describe methods required to prepare data for presentation or synthesis.	No statistical data conversion or imputation was performed; extracted study characteristics and reported metrics were charted narratively/tabled.
METHODS	**13c**	Describe methods used to tabulate or visually display results.	Main Tables 2–4; Supplementary Table S3; Figure 2 for study selection.
METHODS	**13d**	Describe methods used to synthesize results and rationale; if meta-analysis, describe model and software.	Materials and Methods — Synthesis approach. Narrative/thematic synthesis; meta-analysis was not performed because of heterogeneity.
METHODS	**13e**	Describe methods used to explore possible causes of heterogeneity.	Not performed as a statistical subgroup/meta-regression analysis; heterogeneity was characterized narratively by disease setting, EV source, platform, predictor type and validation strategy.
METHODS	**13f**	Describe sensitivity analyses conducted to assess robustness of synthesized results.	Not performed; no quantitative pooled synthesis was undertaken.
METHODS	**14**	Describe methods used to assess risk of bias due to missing results in a synthesis.	Not formally assessed because no quantitative outcome synthesis/meta-analysis was performed; this limitation is reflected

			in the evidence interpretation.
METHODS	**15**	Describe methods used to assess certainty/confidence in the body of evidence.	No GRADE-style certainty assessment was performed. Study-level methodological limitations were evaluated using a PROBAST-informed framework where applicable.
RESULTS	**16a**	Describe results of the search and selection process from records identified to studies included, ideally using a flow diagram.	Results; Figure 2 (PRISMA 2020 flow diagram): 30 identified/screened, 21 excluded, 9 sought/assessed, 0 not retrieved, 0 full-text excluded, 9 included; Supplementary Table S4.
RESULTS	**16b**	Cite studies that might appear eligible but were excluded and explain why.	No formal full-text report was excluded (n=0), so there is no apparently eligible full-text exclusion requiring citation. Supplementary reconciliation exclusions are reported separately.
RESULTS	**17**	Cite each included study and present its characteristics.	Supplementary Table S3; main evidence tables and Results/Discussion.
RESULTS	**18**	Present risk-of-bias assessments for each included study.	Table 4; Supplementary Table S2.
RESULTS	**19**	For all outcomes, present study-level summary statistics/effect estimates and precision where appropriate.	Study-reported diagnostic/classification metrics are presented in the main text and Supplementary Table S3 where available. Conventional effect estimates/precision were not uniformly applicable to this heterogeneous scoping evidence map.
RESULTS	**20a**	For each synthesis, briefly summarise characteristics and risk of bias among contributing studies.	Sections 3.3–3.4; Table 4; Supplementary Tables S2–S3.
RESULTS	**20b**	Present results of all statistical syntheses conducted.	Not applicable: no meta-analysis or other pooled statistical synthesis was

			conducted.
RESULTS	**20c**	Present results of investigations of possible causes of heterogeneity.	Not applicable as a formal statistical analysis; heterogeneity is discussed narratively.
RESULTS	**20d**	Present results of sensitivity analyses.	Not applicable; no sensitivity analysis was conducted.
RESULTS	**21**	Present assessments of risk of bias due to missing results for each synthesis assessed.	Not applicable: no formal reporting-bias assessment was conducted because no quantitative pooled synthesis was performed.
RESULTS	**22**	Present assessments of certainty/confidence in the body of evidence.	No formal certainty grading was performed; study-level methodological limitations and validation gaps are reported instead.
DISCUSSION	**23a**	Provide a general interpretation of results in the context of other evidence.	Discussion.
DISCUSSION	**23b**	Discuss limitations of the evidence included in the review.	Discussion; Limitations.
DISCUSSION	**23c**	Discuss limitations of the review processes used.	Limitations: single primary bibliographic database, supplementary search limitations, no prospective registration/protocol, and related reproducibility constraints.
DISCUSSION	**23d**	Discuss implications for practice, policy and future research.	Discussion; Future Directions; Conclusions.
OTHER	**24a**	Provide registration information, or state that the review was not registered.	Materials and Methods — Protocol and registration: review was not prospectively registered.
OTHER	**24b**	Indicate where the protocol can be accessed, or state that a protocol was not prepared.	Materials and Methods — Protocol and registration: no review protocol was deposited before conduct of the review. No publicly accessible prospective protocol is available, and the archived materials do not document a prospectively prepared protocol.
OTHER	**24c**	Describe and explain	Not applicable: there was
