# Supplementary material for: Artificial Intelligence-Guided Extracellular Vesicle Platforms for Diagnosis and Treatment of Chronic Kidney Disease: A Scoping Review and Future Directions
Source: Medicina (Kaunas). 2026 Sep 28;62(10):1878. doi: 10.3390/medicina62101878 (PMC13706948; doi:10.3390/medicina62101878)
Supplement: Supplementary file 1 [file medicina-62-01878-s001.zip › File S2 PRISMA 2020 Line-by-Line Audit.pdf]

PRISMA 2020 Line-by-Line Audit

medicina-4534164 | audited against the 27-item PRISMA 2020 checklist and sub-items

Audit conclusion: The manuscript now reports the applicable PRISMA 2020 elements and uses a clean PRISMA 2020 flow diagram. Three process-reporting items (8, 9 and 11) remain only partially compliant because the archived review materials do not permit reliable reconstruction of the number/independence of screeners, data extractors, or risk-of-bias appraisers. These facts must not be invented; the limitation is explicitly disclosed in the manuscript.

Item	Topic	Audit status	Evidence / action
1	Title	Compliant	Title explicitly identifies a scoping review.
2	Abstract	Compliant for core scoping-review reporting	Structured abstract now states design, PubMed/MEDLINE source and date, eligibility focus, non-registration, nine-study result, limitations and conclusions. PRISMA-for-Abstracts intervention-specific elements are not all applicable.
3	Rationale	Compliant	Introduction explains CKD/EV/AI rationale and evidence gap.
4	Objectives	Compliant	Explicit formal scoping question in Methods; objectives also framed in Introduction.
5	Eligibility criteria	Compliant	Methods specifies included/excluded report types and evidence grouping.
6	Information sources	Compliant for formal evidence set; contextual route explicitly non-systematic	Formal evidence source is PubMed/MEDLINE, searched 7 Sep 2026. Contextual literature is now clearly labelled non-systematic and excluded from the PRISMA denominator.
7	Search strategy	Compliant for formal evidence set	Full PubMed/MEDLINE search string and date/limits are reported.
8	Selection process	Partially compliant - factual archival limitation	Screening stages and no automation are reported, but archived materials do not permit reliable reconstruction of the number of screeners or whether screening was independent. Manuscript now states this explicitly rather than inventing a procedure.
9	Data collection process	Partially compliant - factual archival limitation	Extraction domains and no automated extraction are reported, but number/independence of extractors cannot be reconstructed. Explicitly disclosed.
10a	Outcomes sought	Compliant for scoping review	Diagnosis, prognosis, biomarker discovery and related computational outcomes are defined.
10b	Other variables	Compliant	Population, EV source/pre-analytics, predictors, AI method, validation, performance, calibration/utility and translation variables are specified.
11	Risk-of-bias methods	Partially compliant - factual archival limitation	PROBAST/PROBAST+AI method and domains are reported, but the number/independence of appraisers cannot be reconstructed. Explicitly disclosed.
12	Effect measures	Not applicable as uniform measure	No common effect measure was prespecified because evidence was heterogeneous; study-reported performance metrics are mapped narratively.

13a	Eligibility for syntheses	Compliant	Nine-study formal evidence set is separated from contextual literature.
13b	Data preparation	Compliant / not required	No conversion or imputation was performed; study characteristics and reported metrics were charted.
13c	Tabulation/display	Compliant	Main tables, Supplementary Table S3 and Figure 2 are identified.
13d	Synthesis methods	Compliant	Narrative/thematic synthesis; rationale for no meta-analysis is stated.
13e	Heterogeneity exploration	Not applicable as statistical analysis	Heterogeneity characterized narratively; no subgroup/meta-regression.
13f	Sensitivity analysis	Not applicable	No quantitative pooled synthesis.
14	Reporting-bias assessment	Not applicable / transparently not performed	No formal missing-results/reporting-bias assessment because no pooled quantitative synthesis.
15	Certainty assessment	Not performed / transparently reported	No GRADE-style certainty grading; study-level methodological appraisal used instead.
16a	Study selection results	Compliant	Clean PRISMA 2020 flow diagram reports 30 identified/screened, 21 excluded, 9 sought/assessed, 0 not retrieved, 0 full-text excluded, 9 included.
16b	Excluded apparently eligible studies	Not applicable to formal full-text set	No full-text report was excluded (n=0). Supplementary reconciliation exclusions are reported separately and are not presented as formal full-text exclusions.
17	Study characteristics	Compliant	Each included study is cited and summarized in Supplementary Table S3/main evidence tables.
18	Risk of bias in studies	Compliant	Study-level/domain-level judgments in Table 4 and Supplementary Table S2.
19	Individual-study results	Compliant where applicable	Study-reported diagnostic/classification metrics are presented; conventional effect estimates/precision are not uniformly applicable to the scoping evidence map.
20a	Synthesis characteristics/ROB	Compliant	Narrative synthesis and ROB summary in Results/Table 4/Supplement.
20b	Statistical synthesis results	Not applicable	No meta-analysis/pooled statistical synthesis.
20c	Heterogeneity investigation results	Not applicable	No formal statistical heterogeneity investigation.
20d	Sensitivity-analysis results	Not applicable	No sensitivity analysis.
21	Reporting biases results	Not applicable	No formal reporting-bias assessment.
22	Certainty results	Not applicable	No formal certainty grading.
23a	Interpretation	Compliant	Discussion interprets findings in context.
23b	Evidence limitations	Compliant	Evidence limitations discussed.
23c	Review-process limitations	Compliant	Single formal database, non-systematic contextual route, archival reviewer-process limitations, no registration/protocol are disclosed.
23d	Implications	Compliant	Practice/translation and future research implications are discussed.
24a	Registration	Compliant	Explicitly states review was not prospectively registered.

24b	Protocol	Compliant	Explicitly states no protocol was deposited/prepared prospectively.
24c	Amendments	Not applicable	No prospective registration/protocol existed; methods/reporting formalization during revision is disclosed.
25	Support	Compliant	Funding statement now specifies no external financial or non-financial support and no funder/sponsor role.
26	Competing interests	Compliant	Conflict-of-interest declaration present.
27	Availability of materials	Compliant	Data Availability Statement points to supplementary screening, study-characterization and ROB materials; no new primary dataset/code generated.

Source: PRISMA 2020 checklist and expanded checklist (Page MJ et al. [59], BMJ 2021;372:n71), CC BY 4.0. This audit preserves the scoping-review design and distinguishes non-applicable items from procedures that were not documented.
