# Supplementary material for: Artificial Intelligence-Guided Extracellular Vesicle Platforms for Diagnosis and Treatment of Chronic Kidney Disease: A Scoping Review and Future Directions
Source: Medicina (Kaunas). 2026 Sep 28;62(10):1878. doi: 10.3390/medicina62101878 (PMC13706948; doi:10.3390/medicina62101878)
Supplement: Supplementary file 1 [file medicina-62-01878-s001.zip › Supplementary Material_AIVEV CKD_FINAL.pdf]

Supplementary Material

Artificial Intelligence-Guided Extracellular Vesicle Platforms for Diagnosis and Treatment of Chronic Kidney Disease: A Scoping Review and Future Directions

Kumar Digvijay, Henrik Birn, Claudio Ronco

This supplement contains Supplementary Table S1 (PRISMA-ScR reporting checklist), Supplementary Table S2 (domain-level PROBAST-informed risk-of-bias judgments, with citation numbers aligned to the manuscript reference list), Supplementary Table S3 (the nine studies retained for qualitative synthesis), and Supplementary Table S4 (screening reconciliation summary). A completed PRISMA 2020 checklist is supplied as a separate reporting file.

Note on reference numbering: citation numbers in Supplementary Tables S2–S3 are aligned to the final revised manuscript reference list.

Supplementary Table S1. PRISMA-ScR reporting checklist

The checklist follows the published PRISMA-ScR framework (20 essential items and 2 optional critical-appraisal items). Locations refer to sections, figures and tables in the final manuscript and this supplement. PRISMA-ScR is the design-specific reporting framework for this scoping review; the separate completed PRISMA 2020 checklist provides an additional reporting cross-check.

Item	PRISMA-ScR item	Requirement	Where reported	Status
1	Title	Identifies the report as a scoping review.	Title	Yes
2	Structured summary	Background, objectives, eligibility criteria, evidence sources, charting, results, conclusions.	Abstract	Yes
3	Rationale	Explains the rationale and why a scoping approach is appropriate.	Introduction; Methods	Yes
4	Objectives	States the review question/objectives and scope.	Methods, Section 2	Yes
5	Protocol and registration	States whether a protocol exists and where it can be accessed.	Methods, Section 2	Yes — no prospectively registered protocol
6	Eligibility criteria	Defines eligibility criteria and exclusions.	Methods, Section 2	Yes
7	Information sources	Reports PubMed/MEDLINE, citation tracking, search date and supplementary identification route.	Methods, Section 2	Yes
8	Search	Provides the complete PubMed/MEDLINE search concepts/string.	Methods, Section 2	Yes
9	Selection of sources of evidence	Describes screening and full-text selection.	Methods, Section 2; Figure 2	Yes
10	Data charting process	Describes extraction/charting domains and narrative synthesis.	Methods, Section 2	Yes
11	Data items	Defines population, EV source, pre-analytics, predictors, AI method, outcomes, validation, metrics, calibration/utility and translation variables.	Methods, Section 2	Yes
12*	Critical appraisal of individual sources	Describes PROBAST appraisal and rationale.	Methods, Section 2; Section 3.4.3; Table 4; Supplementary Table S2	Yes (optional item)
13	Synthesis of results	Explains narrative/thematic synthesis rather than quantitative pooling.	Methods, Section 2; Results/Discussion	Yes
14	Selection of sources	Reports numbers	Figure 2; Methods,	Yes

	of evidence	screened, assessed and included, and reasons for audit exclusions.	Section 2; Supplementary Table S4	
15	Characteristics of sources of evidence	Provides study characteristics and citations in evidence tables.	Tables 2–4; Supplementary Table S3	Yes
16*	Critical appraisal within sources	Presents domain-level appraisal data.	Table 4; Supplementary Table S2	Yes (optional item)
17	Results of individual sources of evidence	Reports representative study-level findings, validation and performance.	Tables 2–4; Sections 3.3–3.4; Supplementary Table S3	Yes
18	Synthesis of results	Maps direct EV–AI evidence, methodological precedents and translational gaps.	Sections 3.3–4.3; Tables 2–6	Yes
19	Summary of evidence	Summarizes the mapped evidence and implications.	Sections 4.2–5	Yes
20	Limitations	Discusses limitations of the review and evidence base.	Section 4.2	Yes
21	Conclusions	Provides an evidence-weighted interpretation and implications.	Section 5	Yes
22	Funding	Reports funding for the review/included evidence as applicable.	Declarations / Funding statement	Yes

Supplementary Table S2. Domain-level PROBAST-informed risk-of-bias judgments for all nine retained EV/EV-associated reports and selected non-EV methodological comparators

Ratings summarize the four original PROBAST domains — participants, predictors, outcome and analysis — using the standard categories Low, High, or Unclear. Because several retained reports are exploratory biomarker-discovery studies rather than formal clinical prediction-model development studies, these judgments are explicitly PROBAST-informed. All nine retained reports containing predictive, classification, or ML-based biomarker analyses are included. Three non-EV comparator studies used in the manuscript to illustrate methodological standards are listed separately within the same table.

Primary study	Evidence type / role	Participants	Predictors	Outcome	Analysis	Overall ROB / applicability
Bienaimé et al. 2023 [24]	Contextual non-EV comparator; primarily an assay-validation study with an exploratory prediction analysis	Low	Low	Low	High	High — exploratory LASSO analysis with repeated internal resampling; no independent external validation.
Ferguson et al. 2022 [37]	Contextual non-EV comparator	Low	Low	Low	Low	Low — large development and independent external-validation cohorts; calibration assessed.
Mendapara 2024 [50]	Contextual non-EV genomic comparator	Unclear	Low	Low	High	High — high-dimensional feature selection relative to sample size despite independent GEO evaluation.
Burrello et al. 2023 [39]	Retained; direct serum/urine EV prediction study	Low	Low	Low	High	High — n=58, 37 EV antigens, extensive exploration of 616 candidate models and leave-one-out validation.

Zhao et al. 2026 [40]	Retained; direct uEV RNA diagnostic model	Low	Low	Low	Low	Low — independent time-separated test cohort, calibration and decision-curve analysis reported; sample size remains limited for transportability.
Zhang H et al. 2025 [41]	Retained; direct uEV RNA biomarker/ML study	Low	Low	Low	High	High — small discovery cohort with high-dimensional RNA screening; candidate RNAs validated independently, but the ML feature-selection stage remains vulnerable to overfitting.
Zhang N et al. 2026 [42]	Retained; direct uEV metabolomics/ML study	Low	Low	Low	High	High — 51 SLE participants for LN/non-LN comparison with high-dimensional metabolomics and RF/RFECV; no independent external validation.
Wang et al. 2025 [44]	Retained; serum exosome metabolomics/ML study	Low	Low	Low	High	High — 91 participants, 586 metabolites, multiple feature-selection algorithms; no independent external validation.

Song et al. 2025 [45]	Retained; DKD ML study with urinary-exosome substudy	Low	Low	Low	High	High — classifier performance was not a purely EV-derived model; EV measurements formed a supporting substudy.
Zhao et al. 2023 [46]	Retained; exploratory uEV transcriptomics/ML study	Low	Low	Low	High	High — 24 participants with high-dimensional transcriptomic discovery and no independent clinical validation.
Yang et al. 2025 [47]	Retained; EV-associated computational biomarker study, not a direct clinical EV assay	Unclear	Low	Low	High	High — tissue/public-dataset derivation, extensive computational feature selection; applicability to direct EV testing is limited.
Bruschi et al. 2021 [48]	Retained; mesothelial exosome proteomics/ML study	High	Low	Low	High	High — extremely small FSGS/non-FSGS groups with high-dimensional proteomics and no independent validation.

a Zhao et al. reported calibration curves (with optimism-bias correction) and decision-curve analysis in addition to discrimination metrics; the analysis domain and overall risk-of-bias judgment were therefore rated Low. The independent time-separated test cohort strengthens the evaluation, although broader external validation is still needed to establish transportability.

Interpretive note. External validation was considered separately from risk of bias. Absence of an external cohort was not treated as an automatic high-risk judgment in isolation; however, small sample size, high-dimensional feature selection, incomplete separation of model development from performance estimation, and indirect EV ascertainment contributed to the analysis or applicability judgments where relevant. Bienaimé et al. is characterized primarily as an assay-validation study with an exploratory prediction analysis.

Key references: Moons KGM, et al. PROBAST. Ann Intern Med. 2019;170:W1–W33. doi:10.7326/M18-1377. Moons KGM, et al. PROBAST+AI. BMJ. 2025;388:e082505. doi:10.1136/bmj-2024-082505.

Supplementary Table S3. Studies retained for qualitative synthesis (n = 9)

All nine reports identified by the archived PubMed/MEDLINE screening pass (30 records screened; 9 assessed at full text; 9 retained) are listed below. Reference numbers correspond to the final revised manuscript reference list. The formal evidence set supports the diagnostic, prognostic, biomarker-discovery, and EV-associated computational synthesis; engineering, PBPK, manufacturing, federated-learning, and digital-twin sections in the manuscript are contextual narrative extensions and are not counted in the PRISMA evidence set.

Study (citation)	PMID	Design / cohort	EV source & modality	AI/ML approach	Key reported findings	Evidence role	Where addressed
Zhao Z, et al. 2023. Comput Biol Med 166:107480 [46]	37738894	Case–control; 24 participants (12 biopsy-proven T2DN, 12 T2DM)	uEV (ultracentrifugation); whole-transcriptome mRNA/lncRNA/circRNA microarrays + miRNA NGS	WGCNA, ceRNA network construction, ML classifiers for candidate RNAs	MYO1C and SP100 kidney-confirmed (Nephroseq, scRNA-seq, HPA); 6 candidate RNAs (2 circRNA, 2 miRNA, 2 lncRNA) proposed as T2DN biomarkers	Core	Main text Section 3.4.2; Table 4; Supplementary Table S3
Zhao Z, et al. 2026. JCI Insight 11(5):e194604 [40]	41569692	Diagnostic model; training/validation 33 T2DM + 40 T2DN; independent test 17 T2DN + 20 T2DM	uEV RNA	ML diagnostic model; mechanistic follow-up of MYO1C	Validation AUC 0.877; independent test AUC 0.824; MYO1C most influential feature; calibration and decision-curve analysis reported	Core	Main text Section 3.4.2; Table 4; Supplementary Table S3
Song J, et al. 2025. BMC Endocr Disord [45]	40474205	Case–control, 800 subjects (200 control / 300 T2DM / 300 DKD); uEV substudy 30 DKD / 30 T2DM / 15 control; 46-DKD genotype subset	Urinary exosomes (ultracentrifugation); GSK3β protein and kinase activity + GSK3B tag SNPs	Six ML algorithms compared; XGBoost best (ROC 0.877, accuracy 0.825)	GSK3B rs60393216 and rs1488766 ranked among top predictors; uEV GSK3β correlated with UACR	Supporting	Main text Section 3.4.2; Table 4; Supplementary Table S3
Yang X, et al. 2025. Ren Fail [47]	39957315	Bioinformatics + Mendelian randomization; glomerular tissue datasets GSE96804 (41 DKD / 20 control) and GSE30528 (9 / 13); 121 exosome-related genes	Tissue transcriptomics (not a direct clinical EV assay); EV association confirmed via Vesiclepedia	WGCNA, MR (IVW), XGBoost + RF feature selection, nomogram with calibration and DCA	CMAS and RGS10 identified as EV-associated DKD biomarkers; nomogram AUC 0.930	Supporting computational	Main text Section 3.4.2; Table 4; Supplementary Table S3

Wang ZY, et al. 2025. Curr Med Sci 45(2):231–243 [44]	40019633	Case–control; 91 participants (58 SLE [30 non-renal, 28 LN], 33 healthy controls)	Serum exosomes (ultracentrifugation); LC-MS/MS metabolomics	Random forest, LASSO, SVM-RFE feature selection	Three metabolites (Pro-Asn-Gln-Met-Ser, C24:1 sphingolipid, protoporphyrin IX); combined AUC 1.0 (SLE vs control) and 0.931 (LN vs non-renal SLE)	Supporting	Main text Section 3.4.2; Table 4; Supplementary Table S3
Zhang H, et al. 2025. Clin Kidney J 18(10):sfaf295 [41]	41104259	Discovery 40 LN; validation cohort 143 SLE urine samples	uEV RNA	Three ML algorithms for candidate prioritization; independent RT-qPCR validation	CCR7, LRRN3, RUNDC3A-AS1 and LINC01127 prioritized for active LN	Core	Main text Section 3.4.2; Table 4; Supplementary Table S3
Zhang N, et al. 2026. Front Immunol 17:1741455 [42]	41782855	29 LN, 22 non-renal SLE, 20 healthy controls; single centre	uEV metabolomics (precipitation-based isolation — noted as a pre-analytical caveat)	Random-forest feature prioritization; ROC evaluation of candidate metabolites	Three metabolites (Lyso-Gb1, PE-NMe, PC (20:5/TXB2)) with AUC 0.912, 0.906 and 0.897 for LN vs non-renal SLE	Core	Main text Section 3.4.2; Table 4; Supplementary Table S3
Burrello J, et al. 2023. Nephrol Dial Transplant 38(3):764–777 [39]	36073758	n = 58 kidney-transplant recipients; 426 samples	Serum and urine EV surface markers	Supervised ML with extensive model search; leave-one-out validation	EV signature proposed for prediction of 1-year renal outcome; high analysis-domain concern (Table 4)	Core / methodological	Main text Section 3.4.2; Table 4; Supplementary Table S3
Bruschi M, et al. 2021. Sci Rep 11:20807 [48]	34675284	Pediatric peritoneal dialysis; 6 FSGS vs 6 non-FSGS	Mesothelial exosomes from peritoneal dialysis effluent; LC-MS proteomics	Nonlinear SVM and PLS-DA; WGCNA	Complete group separation (100%); ANXA13 upregulated and TIMP1 downregulated; 40% of 2,490 proteins linked to EMT/fibrosis and TGF-β pathways	High-risk supporting	Main text Section 3.4.2; Table 4; Supplementary Table S3

Supplementary Table S4. Screening reconciliation summary

Reconciliation of the archived 30-record PubMed/MEDLINE export with the seven-row supplementary screening file. The supplementary file contributed no new eligible studies beyond the PubMed export.

Metric	Count / note
Original PubMed/MEDLINE export	30
Supplementary screening file rows	7
Supplementary rows duplicating PubMed records	2 (one identified as PMID 40019633; the second duplicate retained in the archived export)
Supplementary unique rows excluded — author publication	1 (the present review; not cited or counted as evidence)
Supplementary unique rows excluded — invalid/mismatched records	4 (PMIDs 36412984, 33108422, 29844310, 25441920; recorded titles could not be verified against the PubMed records)
Unique records after reconciliation	35
New valid eligible studies contributed by supplementary file	0
Full-text candidates retained from PubMed export	9 (listed in Supplementary Table S3)

Excluded at title/abstract screening (selected examples): PMID 38728235 (exosome-related risk model in renal cell carcinoma — out of CKD scope).
